# Comprehensive assessment of TDP-43 neuropathology data in the National Alzheimer’s Coordinating Center database

**DOI:** 10.1007/s00401-024-02728-8

**Published:** 2024-06-19

**Authors:** Davis C. Woodworth, Katelynn M. Nguyen, Lorena Sordo, Kiana A. Scambray, Elizabeth Head, Claudia H. Kawas, María M. Corrada, Peter T. Nelson, S. Ahmad Sajjadi

**Affiliations:** 1 Department of Neurology, University of California, Irvine, Office 364, Med Surge II Building, Irvine, CA 92697 USA; 2 Institute for Memory Impairments and Neurological Disorders, University of California, Irvine, CA USA; 3 Department of Pathology and Laboratory Medicine, University of California, Irvine, CA USA; 4 Department of Neurobiology and Behavior, University of California, Irvine, CA USA; 5 Department of Epidemiology and Biostatistics, University of California, Irvine, CA USA; 6 Department of Pathology and Laboratory Medicine, University of Kentucky, Lexington, KY USA

**Keywords:** TDP-43, Limbic predominant age-related TDP-43 encephalopathy neuropathologic change, Frontotemporal lobar degeneration, Amyotrophic lateral sclerosis, Hippocampal sclerosis of aging, Dementia, Alzheimer’s disease, National Alzheimer’s coordinating center

## Abstract

TDP-43 proteinopathy is a salient neuropathologic feature in a subset of frontotemporal lobar degeneration (FTLD-TDP), in amyotrophic lateral sclerosis (ALS-TDP), and in limbic-predominant age-related TDP-43 encephalopathy neuropathologic change (LATE-NC), and is associated with hippocampal sclerosis of aging (HS-A). We examined TDP-43-related pathology data in the National Alzheimer’s Coordinating Center (NACC) in two parts: (I) availability of assessments, and (II) associations with clinical diagnoses and other neuropathologies in those with all TDP-43 measures available. Part I: Of 4326 participants with neuropathology data collected using forms that included TDP-43 assessments, data availability was highest for HS-A (97%) and ALS (94%), followed by FTLD-TDP (83%). Regional TDP-43 pathologic assessment was available for 77% of participants, with hippocampus the most common region. Availability for the TDP-43-related measures increased over time, and was higher in centers with high proportions of participants with clinical FTLD. Part II: In 2142 participants with all TDP-43-related assessments available, 27% of participants had LATE-NC, whereas ALS-TDP or FTLD-TDP (ALS/FTLD-TDP) was present in 9% of participants, and 2% of participants had TDP-43 related to other pathologies (“Other TDP-43”). HS-A was present in 14% of participants, of whom 55% had LATE-NC, 20% ASL/FTLD-TDP, 3% Other TDP-43, and 23% no TDP-43. LATE-NC, ALS/FTLD-TDP, and Other TDP-43, were each associated with higher odds of dementia, HS-A, and hippocampal atrophy, compared to those without TDP-43 pathology. LATE-NC was associated with higher odds for Alzheimer’s disease (AD) clinical diagnosis, AD neuropathologic change (ADNC), Lewy bodies, arteriolosclerosis, and cortical atrophy. ALS/FTLD-TDP was associated with higher odds of clinical diagnoses of primary progressive aphasia and behavioral-variant frontotemporal dementia, and cortical/frontotemporal lobar atrophy. When using NACC data for TDP-43-related analyses, researchers should carefully consider the incomplete availability of the different regional TDP-43 assessments, the high frequency of participants with ALS/FTLD-TDP, and the presence of other forms of TDP-43 pathology.

**Supplementary Information:**

The online version contains supplementary material available at 10.1007/s00401-024-02728-8.

## Introduction

TAR DNA-binding protein of 43 kDa (TDP-43) proteinopathy was first discovered in the context of frontotemporal lobar degeneration (FTLD) and amyotrophic lateral sclerosis (ALS), where a substantial number of participants presented at death with ubiquitinated inclusions but without a tauopathy which might have explained their clinical presentation. These ubiquitinated inclusions were eventually shown to comprise TDP-43 protein [5, 65], which are found in around 95% of patients with ALS and around half of patients with FTLD (FTLD-TDP) [48]. Physiological TDP-43 has a variety of functions, including regulation of mRNA and DNA repair, protein synthesis, and lysosomal function [21]. Fused in Sarcoma (FUS) can also cause both ALS and FTLD and shares similarities with TDP-43 as both proteins serve a role in stabilizing RNA and have similar domain structures [48]. A variety of tauopathies account for approximately the other half of FTLD cases [48].

More recently, TDP-43 inclusions were found to occur in the context of advanced age and classified using the term limbic-predominant age-related TDP-43 encephalopathy neuropathologic change (LATE-NC) [62]. TDP-43 inclusions have also been identified in a variety of other conditions such as chronic traumatic encephalopathy (CTE) [66] and corticobasal degeneration (CBD) [76]. A high percentage of individuals both with FTLD-TDP and LATE-NC also harbor hippocampal sclerosis of aging (HS-A) [60], the neuropathologic finding of disproportionate cell loss and gliosis in the subiculum and/or cornu ammonis 1 (CA1) regions.

While the presence of TDP-43 is common across the above-mentioned pathological conditions, their clinical and demographic characteristics differ widely, with ALS and FTLD occurring at younger ages (usually 50–70 years) and with pronounced frontotemporal atrophy patterns [59], whereas LATE-NC occurs at older ages (usually 80 years and older) with predominantly medial temporal atrophy patterns [10, 36, 80, 83, 84]. LATE-NC appears to be particularly prevalent in the oldest-old and relevant to cognitive decline in this group [56, 77]. Additionally, ALS and FTLD pathologies are generally associated with specific clinical syndromes, namely primary progressive aphasia (PPA) [55], behavioral variant of frontotemporal dementia (bvFTD) [15, 33], and motor neuron disease in ALS [29]. LATE-NC, on the other hand, is more commonly associated with an amnestic syndrome similar to that associated with Alzheimer’s disease neuropathologic change (ADNC) [61]. Thus, while tied together by TDP-43, the demographic and clinical characteristics of these groups differ significantly.

With the growing recognition of the importance of pathological TDP-43 to dementia and with more widespread routine assessment of TDP-43 at neuropathology centers, the National Alzheimer’s Coordinating Center (NACC) included assessment of TDP-43 in the neuropathology version 10 form (v10) [12, 54] used by the national Alzheimer’s Disease Centers (ADCs) since 2014. NACC neuropathology v10 forms and onwards include fields for assessment of TDP-43 inclusions for different regions, including the spinal cord, amygdala, hippocampus, entorhinal cortex or inferior temporal cortex (EC/ITC), and neocortex. The presence of FTLD-related TDP-43 is a separate category in these pathology forms, and ALS-related TDP-43 is also captured by another variable assessing motor neuron inclusions. Assessment of HS-A was also updated in the v10 forms from a more general scoring for medial temporal lobe sclerosis to a specific assessment of severe neuronal loss and gliosis in CA1 and/or subiculum. NACC data enables researchers to examine associations across large sample sizes by pooling participants across the multiple contributing ADCs. Studies that have leveraged the large samples from NACC data have documented findings focused on single pathological entities such as LATE-NC [13], incorporated LATE-NC into associations of pathologies with cognition and cognitive decline [40, 75], examined differences across LATE-NC patients split by HS-A [27] or AD pathology [26], and assessed similarities and differences between participants with LATE-NC and FTLD-TDP [74]. Given the wealth of data on these TDP-43 related pathologies captured in the pathology forms and the large number of contributing centers and participants in the NACC database, this database and categorization system is a critical tool for better understanding TDP-43 related pathologies.

In this study we aimed evaluate comprehensively the TDP-43 related data in NACC. We performed our study in two parts. For Part I, we documented availability of TDP-43-related pathology data available in NACC. In Part II, in those with all TDP-43-related measures available, we examined the regional distribution, demographic characteristics, clinical symptoms, and co-occurrence of other neuropathologies, for the TDP-43 categories of FTLD-TDP, ALS-TDP, LATE-NC, as well as for TDP-43 related to other pathologies.

## Materials and methods

### Participants and demographic variables

We used information from participants in the NACC database from September 2005 through March 2023. Because certain ADCs tend to be enriched for particular clinical presentations, such as AD or FTD [25], participants with these conditions are over-represented in the NACC database with respect to the general population. To provide a sense of the relative rates of follow-up and autopsy by cognitive diagnosis, we examined rates of follow-up and autopsy by last available presumptive clinical etiologic diagnosis. We used presumptive clinical etiologic diagnosis as, for many neurodegenerative conditions, this is the only observable manifestation of the underlying pathology, and is likely to affect follow-up and autopsy rates through a variety of mechanisms. We examined the proportion of participants lost to follow-up, as well as the proportion of participants who died, who were assessed at autopsy, and who were assessed for TDP-43, by their last available presumptive etiologic diagnosis. We grouped the last available presumptive etiologic diagnosis into eight different categories: (1) AD; (2) an FTLD category composed of the “FTLD Other” and “FTLD motor neuron disease” designations to capture participants most likely to have ALS-TDP or FTLD-TDP; (3) a category consisting of those with either CBD or primary progressive supranuclear palsy (PSP) to capture FTD conditions more likely to be associated with a tauopathy rather than TDP-43; (4) Lewy body dementia; (5) vascular dementia; (6) an “Other” impairment condition that contained a wide variety of presumptive etiologic diagnoses such as other neurodegenerative, medical, or psychiatric conditions; (7) a category for those without impairment at their last available visit; and (8) a category for participants where an presumptive etiologic diagnosis at the last visit was not available.

**Fig. 1:**
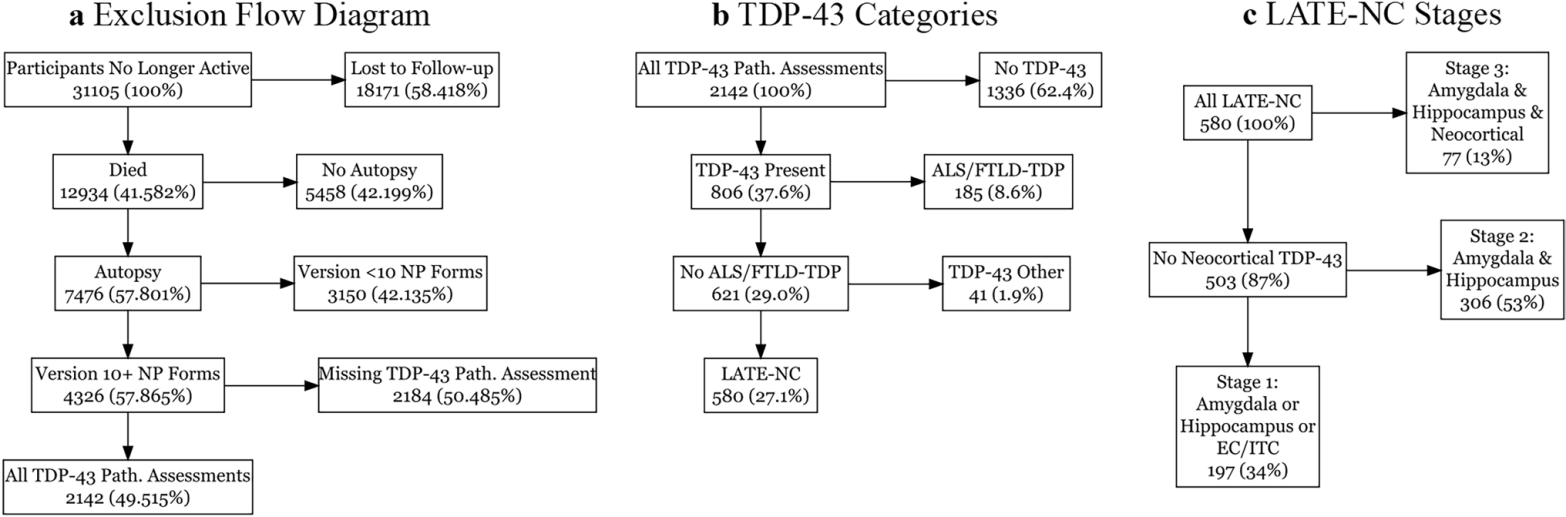
Flow diagrams for **a** the inclusion/exclusion of participants **b** assigning of TDP-43 categories and **c** LATE-NC stages. Numbers are shown as: N (percentage of previous) for (**a**) and *N* (percentage of total) in (**b**, **c**). *NP* neuropathology. *Path.* Pathology, *ALS/FTLD-TDP* amyotrophic lateral sclerosis or frontotemporal lobar degeneration with TDP-43 pathology, *LATE-NC* limbic-predominant age-related TDP-43 encephalopathy neuropathologic change, *EC/ITC* entorhinal cortex/inferior temporal cortex

The majority of our examinations for Part I were limited to participants who had version 10 or later neuropathology [12] and Uniform Data Set (UDS) [9] assessments available, as shown in Fig. 1a. We used demographic, clinical, and cognitive information from the last visit prior to death. For demographic information, we used age at death, sex, years of education, and interval between the last visit and death. We also report the frequency of an apolipoprotein E4 (APOE4) allele using information from the NACC genetic forms. We used information on reported hereditary FTD mutation, which was available as “Yes” or “No/unknown”. We used dementia diagnosis and Clinical Dementia Rating (CDR® Dementia Staging Instrument) sum-of-the-boxes (CDR-SB) as measures of overall cognition. We used information from the clinician diagnoses for primary progressive aphasia (PPA) and behavioral variant of frontotemporal dementia (bvFTD). PPA and bvFTD assessments were available for all UDS versions, and thus allowed for maximal use of neuropathology data. We also used the clinical presumptive etiologic diagnosis of Alzheimer’s disease as a measure for clinical syndromes of Alzheimer’s type (i.e. clinical AD). Lastly, we used the NACC variable for the different ADCs, which are numeric IDs that are randomly generated to maintain site anonymity but which enable examination of the individual measures by center. Other clinical assessment fields relating to FTD and ALS, such as clinician assessment of findings related to, and presumptive etiologic diagnosis of, FTD motor neuron disease, motor changes related to ALS, and clinician assessment of FTD not otherwise specified, were available for version 3 and onward UDS data [11], and as such were less widely available and were not examined in this study.

### TDP-43 related pathology information

TDP-43 related pathology information is coded in the NACC database through multiple variables. There is the assessment for presence of TDP-43 immunoreactive inclusions in various regions, including the spinal cord (NACC variable NPTDPA), amygdala (NPTDPB), hippocampus (NPTDPC), entorhinal cortex and/or inferior temporal cortex (EC/ITC, NACC variable NPTDPD) and neocortex (NPTDPE). We primarily focused on the TDP-43 scores for the brain regions, as these were more widely available and relevant towards disambiguating some of the neuropathologies with greater regional overlap such as LATE-NC and FTLD-TDP. However, we also report some analyses examining spinal cord TDP-43 inclusions using the more limited sample with this measure available. There is a separate variable for denoting presence of FTLD-related TDP-43 (NPFTDTDP). ALS-related pathology findings are captured by a more general variable (NPALSMND) that classifies motor neuron inclusions into different categories: TDP-43 inclusions, fused in sarcoma (FUS) inclusions, superoxide dismutase-1 (SOD1) inclusions, other inclusions, or no specific inclusions. Hippocampal sclerosis of aging (HS-A) is coded in the NACC variable NPHIPSCL, which classifies HS-A as present unilaterally, bilaterally, or present without laterality assessed; for this study we used presence of HS-A, regardless of laterality, as our measure of interest, as many centers only examine one hemisphere histopathologically. We also evaluated information on the TDP-43 antibody used (NPTDPAN) which indicated whether the antibody was phospho-specific, non-phospho-specific, or “other”.

We classified participants into several groups based on their TDP-43 pathology data. Those without brain TDP-43, and without FTLD-TDP or ALS-TDP designations, were classified in a “No TDP-43” group, irrespective of other pathologies present; thus, while these participants did not have TDP-43, they could have other rare pathologies, such as forms of FTLD-related tau, or more common pathologies such as ADNC or Lewy bodies. For our TDP-43 groups we implemented the updated (2023) LATE-NC criteria [63]. We classified anyone with ALS-TDP or FTLD-TDP indicated on their pathology forms as ALS-TDP and/or FTLD-TDP, irrespective of which other pathologies might be present. There are other pathologies that are associated with TDP-43 which should not be considered LATE-NC, which we chose to group in an “Other TDP-43” group. We selected the pathologies we considered could appear in NACC and be associated with non-LATE-NC and non-ALS/FTLD-TDP, TDP-43: corticobasal degeneration (CBD) [76], Parkinsonism of Guam and Kii [30], chronic traumatic encephalopathy (CTE) [66], traumatic brain injury (TBI) [1], and Huntington disease [70]. Additionally, the updated LATE-NC criteria advises against assigning LATE-NC to those with TDP-43 that also have an FTD-related mutation even if the case has not been designated as ALS-TDP or FTLD-TDP by a neuropathologist. If a participant had TDP-43 in the brain and was indicated as having an FTD-related mutation, either in visit forms or as assessed at autopsy, and were not assigned ALS-TDP or FTLD-TDP at autopsy, these were also included in the “Other TDP-43” group. We also classified those with TDP-43 and autosomal dominant AD (ADAD) mutations, and without ALS/FTLD-TDP, as assessed at a visit or at autopsy, in the “Other TDP-43” group. Lastly, while isolated TDP-43 at the level of the amygdala or hippocampus is allowed within the updated LATE-NC criteria, isolated TDP-43 in the neocortex without both amygdala and hippocampal TDP-43 is not; as such we have included participants with neocortical TDP-43, but missing TDP-43 in either the hippocampus or the amygdala, in the “Other TDP-43” group. While this “Other TDP-43” group is heterogeneous since it captures TDP-43 related to multiple conditions, we opted to include this catch-all group for non-ALS/FTLD-TDP and non-LATE-NC TDP-43, in order to account for all occurrences of TDP-43 in the available data. Only participants with brain TDP-43 who did not have ALS or FTLD-TDP or the aforementioned TDP-43 related pathologies were then classified as LATE-NC. In the data used, ALS-TDP often co-occurred with FTLD-TDP, and so we opted to group ALS-TDP and FTLD-TDP into a single entity of ALS/FTLD-TDP, which is consistent with the concept that these two pathologies exist on an ALS/FTLD spectrum [19, 49]. In Fig. 1b we show diagrams for how the TDP-43 categories were assigned to participants with all TDP-43 related information available. HS-A, while occurring more often in participants with TDP-43, can be present in TDP-43 negative participants as well, and as such is an entity that is separate, though strongly related, to TDP-43.

For the regional TDP-43 data, we report distributions of TDP-43 inclusions across all regions for each of the TDP-43 categories (ALS/ FTLD-TDP, LATE-NC and Other TDP-43). We applied the updated LATE-NC staging criteria as follows: stage 1 was defined by isolated TDP-43 in the amygdala, hippocampus, or EC/ITC, stage 2 was defined by TDP-43 presence in both the amygdala and hippocampus, and stage 3 by TDP-43 in the amygdala, hippocampus, and neocortex. This designation of LATE-NC stages is illustrated in Fig. 1c.

### Other neuropathology findings of interest

In addition to the TDP-43 pathologies listed above, we used information from other pathologies available in NACC data, assessed using standard guidelines [34, 57]. We used presence of FTLD-related tau, Alzheimer’s disease neuropathologic change (ADNC) dichotomized as none/low vs intermediate/high likelihood, cerebral amyloid angiopathy (CAA) as none/mild vs moderate/severe, Lewy bodies (LB) dichotomized as present for any region (brainstem, amygdala predominant, limbic transitional, neocortical, or olfactory bulb), atherosclerosis as none/mild vs moderate/severe, arteriolosclerosis as none/mild vs moderate/severe, and presence of vascular lesions such as infarcts, microinfarcts and hemorrhages. Lastly, we examined gross assessment of hippocampal and cortical atrophy, both as none/mild vs moderate/severe, as well as presence of gross frontal/temporal lobar atrophy, at autopsy.

### Statistical analyses

Part I: First, to evaluate rates of follow-up and autopsy by presumptive clinical etiologic diagnosis, we used data from participants listed as no longer active in NACC, thus presumed to be either lost to follow-up or deceased. We examined the rates of loss to follow-up and rates of autopsy by presumptive etiologic diagnosis categories, and accounting for center using multilevel logistic regression models with availability as the outcome, population level effects for presumptive etiologic diagnosis category, and varying intercept for center. Within those with autopsy data, we report the availability of the TDP-43 related measures by year of death of participants to show the uptake in TDP-43 assessments over time. We then examined within those with NACC v10 neuropathology forms the rates of TDP-43 assessment by presumptive etiologic diagnosis and center using multilevel models. We also report total availability for TDP-43 related pathology measures in participants with v10 + neuropathology forms and display these graphically using Venn diagrams.

Part II: We then limited the sample to participants who had all TDP-43 related pathology information available (ALS-TDP, FTLD-TDP, and all brain regional TDP-43 assessments). We report frequency and regional distribution of TDP-43 pathology for the different TDP-43 groups (ALS-TDP, FTLD-TD, Other TDP-43 and LATE-NC), and display the complete distribution of TDP-43 inclusions across brain regions for the TDP-43 categories using Venn diagrams. We report participant demographic, clinical characteristics, and co-occurring pathologies by the TDP-43 categories. We also report distribution of TDP-43 across brain regions, and compare sub-groups, within the ALS/FTLD-TDP group, split into FTLD-TDP only, ALS-TDP only, and both ALS-TDP and FTLD-TDP. For group comparisons we report Wilcoxon rank sum tests for continuous variables and report Chi-square tests for categorical variables. To examine the associations of the TDP-43 groups with clinical diagnoses and other neuropathologies, we performed logistic regressions with each of the clinical or other neuropathological findings as the outcome, TDP-43 category as the independent variable of interest, adjusting for age at death, sex, years of education, and interval between last visit and death. We also examined these associations with clinical/neuropathology findings using multilevel models with a varying intercept for center. For both group comparisons and logistic regression models, in addition to the comparison to the reference group of those without TDP-43, we also examined differences between the groups (i.e. LATE vs ALS/FTLD-TDP, LATE vs Other, ALS/FTLD-TDP vs Other) using post-hoc tests for the logistic regressions. For all models we performed complete case analyses with respect to the outcome variables, which had relatively limited degrees of missingness as reported in Table 1. To graphically illustrate the relative frequency and overlap of ALS/FTLD-TDP or LATE-NC with ADNC and LB within NACC data, we created a proportional Euler diagram of these pathologies.

**Table 1:** Participant characteristics and co-pathologies by TDP-43 category: LATE-NC, ALS/FTLD-TDP, Other TDP-43, or No TDP-43

Characteristic	Pathology				*P* values		
	No TDP-43, *N* = 1336^*1*^	Other TDP-43, *N* = 41^*1*^	LATE-NC, *N* = 580^*1*^	ALS/FTLD, *N* = 185^*1*^	LATE vs. No TDP-43^*2*^	LATE vs. ALS/FTLD^*2*^	ALS/FTLD vs. No TDP-43^*2*^
Age at death (Years)	78.9 (12.1)	81.0 (12.3)	84.0 (9.5)	71.5 (11.0)	< 0.001	< 0.001	< 0.001
Sex					0.066	0.2	0.9
Female	640 (48%)	15 (37%)	303 (52%)	87 (47%)			
Male	696 (52%)	26 (63%)	277 (48%)	98 (53%)			
Education (Years)	15.8 (3.0)	15.2 (2.5)	15.8 (2.9)	15.8 (2.9)	0.5	> 0.9	0.7
Missing	10	0	3	6			
Interval last visit to death (Years)	2.2 (2.4)	2.4 (3.1)	2.5 (2.6)	1.8 (2.0)	0.048	0.006	0.078
APOE e4					< 0.001	< 0.001	0.12
Absent	732 (61%)	24 (67%)	225 (42%)	108 (68%)			
Present	465 (39%)	12 (33%)	306 (58%)	51 (32%)			
Missing	139	5	49	26			
Hereditary FTD Mutation					0.008	< 0.001	< 0.001
Yes	14 (1.0%)	6 (15%)	0 (0%)	42 (23%)			
No or unknown	1322 (99%)	35 (85%)	580 (100%)	143 (77%)			
TDP-43 antibody					0.002	< 0.001	0.022
Phospho-specific	947 (71%)	31 (76%)	456 (79%)	115 (62%)			
Non-phospho-specific	381 (29%)	10 (24%)	120 (21%)	70 (38%)			
Other	8 (0.6%)	0 (0%)	4 (0.7%)	0 (0%)			
Cognitive status					< 0.001	0.2	< 0.001
Normal	232 (17%)	3 (7.3%)	34 (5.9%)	8 (4.3%)			
MCI/Impaired	159 (12%)	4 (9.8%)	41 (7.1%)	7 (3.8%)			
Dementia	945 (71%)	34 (83%)	505 (87%)	170 (92%)			
CDR-SB	8.6 (6.7)	10.1 (6.0)	10.9 (5.9)	12.3 (5.8)	< 0.001	0.004	< 0.001
Clinical AD					< 0.001	< 0.001	< 0.001
No impairment	232 (17%)	3 (7.3%)	34 (5.9%)	8 (4.3%)			
Not AD	363 (27%)	13 (32%)	70 (12%)	126 (68%)			
AD	741 (55%)	25 (61%)	476 (82%)	51 (28%)			
PPA diagnosis					0.017	< 0.001	< 0.001
Absent	1148 (92%)	35 (90%)	532 (96%)	121 (67%)			
Present	94 (7.6%)	4 (10%)	25 (4.5%)	59 (33%)			
Missing	94	2	23	5			
bvFTD diagnosis					< 0.001	< 0.001	< 0.001
Absent	1221 (91%)	34 (83%)	563 (97%)	110 (59%)			
Present	115 (8.6%)	7 (17%)	17 (2.9%)	75 (41%)			
HS-A					< 0.001	0.3	< 0.001
Absent	1270 (95%)	31 (76%)	425 (73%)	128 (69%)			
Present	66 (4.9%)	10 (24%)	155 (27%)	57 (31%)			
FTLD-Tau					0.001	0.8	0.2
Absent	1044 (78%)	20 (49%)	486 (84%)	153 (83%)			
Present	292 (22%)	21 (51%)	93 (16%)	32 (17%)			
Missing	0	0	1	0			
ADNC					< 0.001	< 0.001	< 0.001
None/Low	485 (37%)	11 (28%)	86 (15%)	145 (80%)			
Intermediate/High	819 (63%)	28 (72%)	489 (85%)	36 (20%)			
Missing	32	2	5	4			
CAA					< 0.001	< 0.001	< 0.001
None/Mild	937 (70%)	28 (68%)	343 (59%)	161 (88%)			
Moderate/Severe	396 (30%)	13 (32%)	235 (41%)	21 (12%)			
Missing	3	0	2	3			
Lewy bodies					< 0.001	< 0.001	< 0.001
Absent	865 (65%)	26 (63%)	279 (48%)	156 (85%)			
Present	468 (35%)	15 (37%)	301 (52%)	28 (15%)			
Missing	3	0	0	1			
Atherosclerosis					< 0.001	< 0.001	0.2
None/Mild	891 (67%)	29 (71%)	329 (57%)	133 (72%)			
Moderate/Severe	432 (33%)	12 (29%)	249 (43%)	51 (28%)			
Missing	13	0	2	1			
Arteriolosclerosis					< 0.001	< 0.001	0.4
None/Mild	661 (51%)	16 (41%)	211 (38%)	95 (54%)			
Moderate/Severe	646 (49%)	23 (59%)	341 (62%)	81 (46%)			
Missing	29	2	28	9			
Infarcts					0.5	< 0.001	0.002
Absent	1142 (86%)	36 (88%)	490 (85%)	175 (95%)			
Present	187 (14%)	5 (12%)	89 (15%)	10 (5.4%)			
Missing	7	0	1	0			
Microinfarcts					0.022	< 0.001	0.002
Absent	1022 (77%)	34 (83%)	417 (72%)	161 (87%)			
Present	308 (23%)	7 (17%)	162 (28%)	24 (13%)			
Missing	6	0	1	0			
Hemorrhages					0.2	0.5	0.14
Absent	1221 (93%)	35 (90%)	533 (94%)	171 (96%)			
Present	95 (7.2%)	4 (10%)	32 (5.7%)	7 (3.9%)			
Missing	20	2	15	7			
Gross hippocampal atrophy					< 0.001	0.078	< 0.001
None/Mild	789 (62%)	18 (49%)	241 (42%)	55 (34%)			
Moderate/Severe	487 (38%)	19 (51%)	329 (58%)	106 (66%)			
Missing	60	4	10	24			
Gross cortical atrophy					0.023	< 0.001	< 0.001
None/Mild	710 (58%)	20 (54%)	290 (52%)	35 (25%)			
Moderate/Severe	519 (42%)	17 (46%)	270 (48%)	105 (75%)			
Missing	107	4	20	45			
Gross lobar atrophy					0.032	< 0.001	< 0.001
Absent	932 (76%)	29 (78%)	452 (81%)	57 (40%)			
Present	295 (24%)	8 (22%)	108 (19%)	85 (60%)			
Missing	109	4	20	43			

*LATE-NC* limbic-predominant age-related TDP-43 encephalopathy neuropathologic change, *ALS/FLTD-TDP* amyotrophic lateral sclerosis or frontotemporal lobar degeneration with TDP-43, *MCI* mild cognitive impairment, *CDR-SB* Clinical Dementia Rating sum-of-the-boxes, *AD* Alzheimer’s disease, *PPA* primary progressive aphasia, bvFTD, behavioral variant of frontotemporal dementia, *HS-A* hippocampal sclerosis of aging, *ADNC* Alzheimer’s disease neuropathologic change, *CAA* cerebral amyloid angiopathy

^*1*^Mean (SD); *n* (%)

^*2*^Wilcoxon rank sum test; Pearson's Chi-squared test

### Software tools

We used a variety of tools and packages within *R* statistical software (*v4.3*). Flow charts for exclusion, TDP-43 category, and LATE-NC stage flow charts were created using the *PRISMAstatement* package in *R*. Logistic regressions were performed using the *glm* function [35]. Multilevel models were performed using generalized linear mixed effect models (logistic regressions) using the *lme4* package [8], with varying intercepts for center. Post-hoc contrasts between the TDP-43 categories were evaluated using the *emmeans* package [46]. Venn diagrams were created using the package *ggVennDiagram* [24]. Participant characteristic tables were generated using the *gtsummary* package [23]. The proportional Euler diagram for the overlap in pathologies was created using the *eulerr* package [44]. Sankey flow diagrams by presumptive etiologic diagnosis were created using SankeyMatic (https://sankeymatic.com/). We report un-adjusted *P*-values.

## Results

### Part I, availability: general patterns of follow-up, autopsy, and TDP-43 assessment

First, to examine rates of follow-up and autopsy by presumptive clinical etiologic diagnosis, we started with 31,105 participants, across 45 different ADCs, who were listed as no longer active in NACC data. Of these, 12,094 (42%) were reported to have been deceased, the rest assumed lost to follow-up. Among those reported deceased, 7476 (58%) were assessed at autopsy. Follow-up and autopsy rates differed by presumptive etiologic diagnosis categories, with participants diagnosed with FTD syndromes (FTLD or CBD/PSP) having higher follow-up and autopsy rates compared to those with a presumptive AD etiologic diagnosis or without cognitive impairment (Supplementary Fig. 1). As expected, those with clinical FTLD had a higher rate of follow-up until death (69%) and higher autopsy rate among those reported deceased (71%), while those with clinical AD had lower follow-up (50%) and autopsy (58%) rates. From multilevel models, presumptive etiologic diagnosis of FTLD was significantly associated with rate of follow-up (OR = 2.0, *P* < 0.001) compared to those with AD. Follow-up rates were also higher for CBD/PSP (OR = 3.5), and LB (OR = 1.9), but lower for vascular dementia (OR = 0.75) and those with other impairments (OR = 0.43), without impairment (OR = 0.26), or with presumptive etiologic diagnosis unavailable (0.35, *P* < 0.001 for all), compared to those with a presumptive etiologic diagnosis of AD. Within participants reported as deceased, autopsy rate was higher in those with presumptive etiologic diagnosis of FTLD (OR = 1.8, *P* < 0.001), CBD/PSP (OR = 1.8, *P* < 0.001), and LB (OR = 1.2, *P* = 0.01), but lower in those vascular dementia (OR = 0.70, *P* < 0.001), no cognitive impairment (OR = 0.63, *P* < 0.001) or cognitive impairment unavailable (OR = 0.71, *P* < 0.001), compared to those with clinical AD. These differences were exacerbated when center effects were not accounted for (data not shown).

**Fig. 2:**
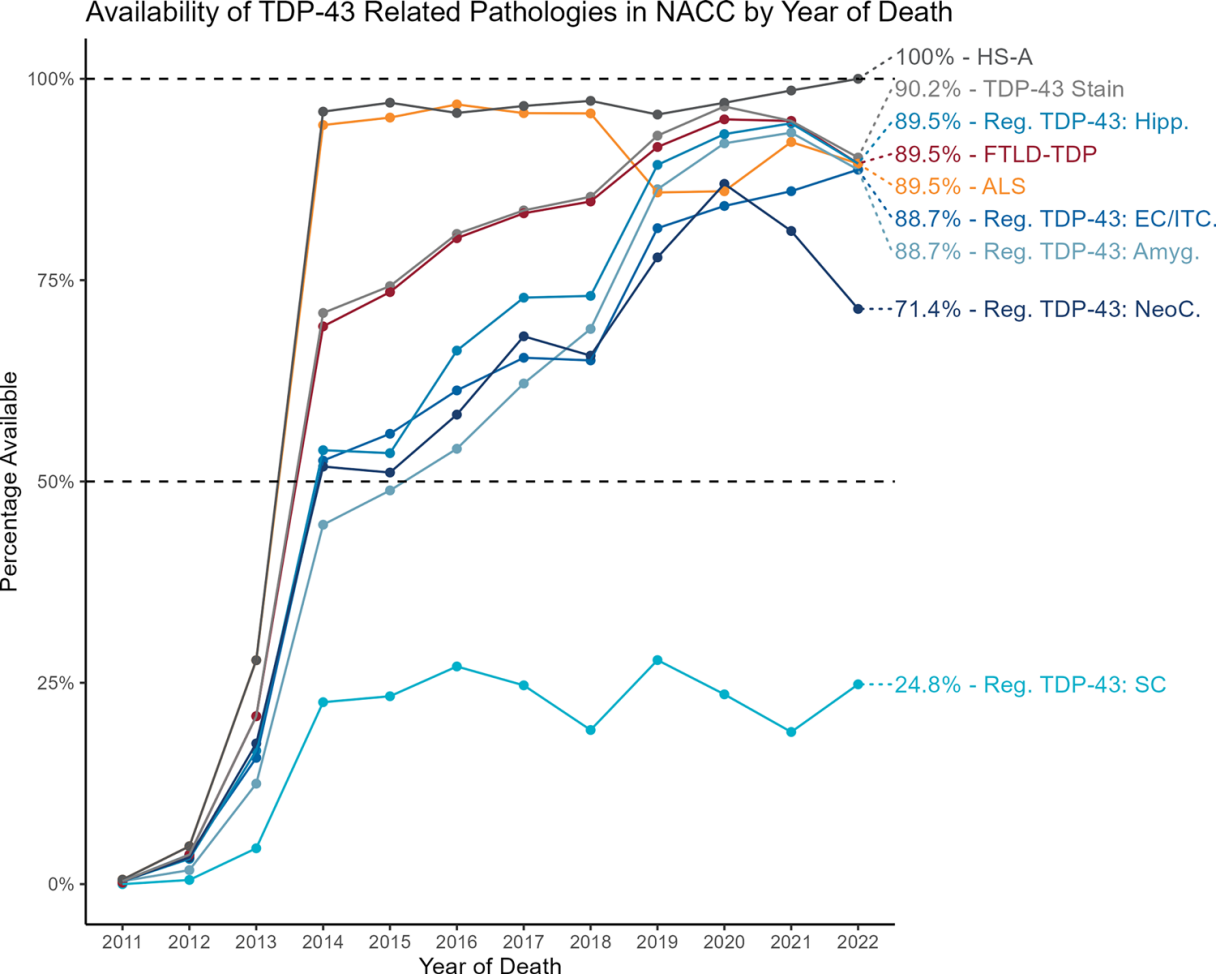
Availability of TDP-43 related pathology measures in NACC database by year of death of participant. *NACC* National Alzheimer’s Coordinating Center, *HS-A* hippocampal sclerosis of aging, *FTLD-TDP* frontotemporal lobar degeneration with TDP-43 pathology, *ALS* amyotrophic lateral sclerosis, *Reg*. regional, *Amyg*. amygdala, *Hipp*. hippocampus, *EC/ITC* entorhinal cortex/inferior temporal cortex, *NeoC*. neocortex, *SC* spinal cord

Then, to examine trends in TDP-43 assessment availability over time, we took data from all participants with autopsy data, consisting of 7476 participants across 39 ADCs. Figure 2 shows the availability of the TDP-43 related measures by year of death. This figures depicts both a transition to NACC pathology version 10 forms occurring with participants who died in 2012 and 2013, followed by increasing availability of various TDP-43 related measures once NACC v10 pathology forms were widely implemented. This figure also shows the availability of the measures for participants who died in 2022 (numbers to right of graph, *N* = 133). Both ALS and HS-A measures began with high availability (both > 90% after 2013), which is expected given that these assessments do not necessarily require TDP-43 specific stains. All participants with FTLD-TDP or regional TDP-43 scores had TDP-43 antibody (NPTDPAN) reported, in contrast with a substantial number of participants with assessment for HS-A (*N* = 686) and ALS (*N* = 645) which did not have a TDP-43 antibody listed. ALS information became slightly less available from 2018 onwards and was available for 90% of participants from 2022, in contrast to HS-A information which remained widely available throughout and was available for all participants who died in 2022. FTLD-TDP and brain regional TDP-43 measures initially saw a slower uptake, but became more widely available with time, with high availability in 2022 for FTLD-TDP (90%) and regional TDP-43 (hippocampal = 90%, amygdala = 89%, EC/ITC = 88%). Availability of the neocortical TDP-43 assessment dropped somewhat in recent years, going from around 90% availability in 2019 to 71% availability for participants who died in 2022. Given the implementation of LATE-NC as a pathological entity and its corresponding staging scheme in 2019 [62], we hypothesized that this decrease in neocortical TDP-43 assessment is likely related to pathologists foregoing this assessment if TDP-43 inclusions were not found in the hippocampus, which we test in the section below.

### Part I, availability: TDP-43 related pathology measures in v10 + neuropathology data

**Fig. 3:**
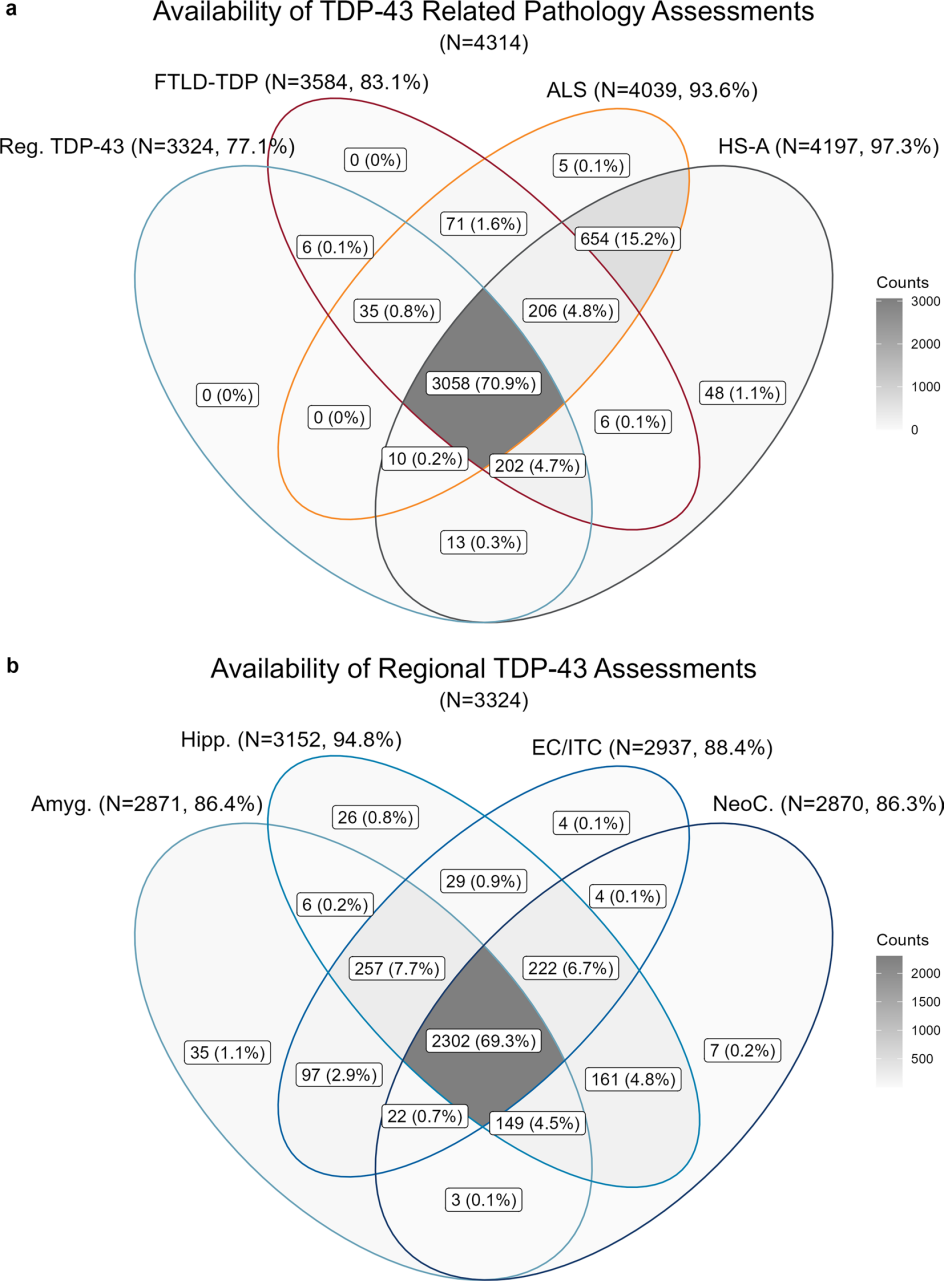
Availability of TDP-43 related pathology measures in National Alzheimer’s Coordinating Center (NACC) participants. **a** Venn diagram of availability across the various TDP-43 pathology measures in participants with version 10 + pathology forms (*N* = 4319). **b** Venn diagram of availability of TDP-43 assessment by region in participants with at least one regional assessment available (*N* = 3324). *NACC* National Alzheimer’s Coordinating Center, *HS-A* hippocampal sclerosis of aging, *FTLD-TDP* frontotemporal lobar degeneration with TDP-43 pathology, *ALS* amyotrophic lateral sclerosis, *Reg*. regional, *Amyg.* amygdala, *Hipp.* hippocampus, *EC/ITC* entorhinal cortex/inferior temporal cortex, *NeoC.* neocortex

We then used data from 4326 participants with v10 (and onwards) pathology forms, derived from 35 ADCs, to assess overall availability of the TDP-43 related measures. FTLD-TDP assessment was available for 3584 (83%) of participants, ALS assessment was available for 4039 (94%), and 3324 participants (77%) had at least one regional TDP-43 assessment available. HS-A assessment was available in 4197 participants (97%). Figure 3a shows the distribution of availability across these TDP-43 related variables. Notably, while a large proportion of participants (3058, 71%) had at least one regional TDP-43, FTLD-TDP, ALS, and HS-A assessments available, a significant proportion only had ALS and HS-A assessments (654, 15%, likely due to the considerations mentioned above relating to not requiring a TDP-43 stain for these measures) or were only missing a regional TDP-43 assessment (206, 5%) or ALS assessment (202, 5%). Figure 3b shows the availability of regional assessments across the amygdala, hippocampus, EC/ITC, and neocortex in those with at least one regional TDP-43 assessment available (*N* = 3324, 77% of total). Of these, 2871 participants (86%) had TDP-43 assessment for the amygdala available, 3152 (95%) had an assessment for the hippocampus, 2937 (88%) had an assessment for the EC/ITC available, 2870 (86%) had an assessment for the neocortex available. 2302 (69%) participants had assessment of TDP-43 inclusions in all four regions, and 2451 (74%) had assessment for TDP-43 in all the LATE-NC staging-relevant regions of the amygdala, hippocampus, and neocortex.

We examined assessment for TDP-43 (TDP-43 staining), and availability of all TDP-43 measures, with regards to last available presumptive etiologic diagnosis, which we show graphically in Supplementary Fig. 2. Participants with clinical FTLD and CBD/PSP were more likely to have been assessed for TDP-43 (FTLD, 97%; CBD/PSP, 93%) compared to other presumptive etiologic diagnoses (80–85%). However, this difference in rates was only statistically significant for FTLD (OR = 2.6, *P* < 0.001). Additionally, within most centers there was no difference for assessing TDP-43 in participants with FTLD compared to those with AD: out of 25 centers with both participants with FTLD and participants with AD, 14 centers had identical rates of TDP-43 staining for these groups. Of the remaining 11 centers, only one center had a significantly higher rate (by Chi-square test) of TDP-43 assessment in participants diagnosed with FTLD (*N* = 30, 97% assessed for TDP-43) compared to participants with AD (*N* = 143, 68% assessed for TDP-43, *P* = 0.001). Availability of all TDP-43 related assessments was much more similar between presumptive etiologic diagnosis, at around 50%. All TDP-43 measures were available for 61% of those with FTLD, 51% of those with CBD/PSP, 47% of those with AD, and slightly lower for those with vascular (42%) and LB (41%) presumptive etiologic diagnoses. In multilevel logistic regression models, which adjust for center, only participants with a presumptive etiologic diagnosis of FTLD were significantly more likely to have all the TDP-43 measures, though this difference in availability was much less pronounced (OR = 1.4, *P* = 0.015).

For those who were assessed for TDP-43 (3625 participants, 84% of total v10 + neuropathology participants), we examined the type of TDP-43 antibody used. For 2237 participants (62%), a phospho-specific TDP-43 antibody was used, 1372 (38%) listed a non-phospho specific antibody, and 16 (0.4%) listed “other”. For those with TDP-43 antibody listed as “other”, five were listed as “ps409/410”, seven as “10,782–2-AP” (one Leica, six Proteintech), three as “polyclonal”, and one as “not specified”. Most of these are phospho-specific. ALS-related inclusions were found present in around 2% of participants with TDP-43 antibody listed, but only in one case out of 645 (0.2%) when TDP-43 antibody was not listed. Of note, this case with ALS but no TDP-43 antibody listed was categorized as having TDP-43 inclusions and thus potentially constitutes an entry error for either the TDP-43 antibody or the type of motor neuron inclusion.

We show the distribution, by ADC, of the availability of TDP-43 measures in Supplementary Fig. 3a, and the presence of these pathologies within those with the measure available in Supplementary Fig. 3b. There was substantial variability, especially in the availability of regional TDP-43 assessments, across centers. We go over these results in more detail in the Supplementary Text. To test our hypothesis that starting in 2019 neuropathology cores of ADCs may have begun foregoing neocortical TDP-43 assessment if no inclusions were found in the hippocampus (in response to LATE-NC staging), we compared the rate of availability of neocortical TDP-43 assessment by presence of TDP-43 in the hippocampus in those with a presumptive etiologic diagnosis of AD (most relevant to LATE-NC), both for participants who died before, and participants who died during or after, 2019. Before 2019, availability rates for neocortical TDP-43 were similar for those without TDP-43 in the hippocampus (638 out of 707, 90%) and those with TDP-43 in the hippocampus (311 out of 340, 92%, Chi-square *P* = 0.5). However, for participants who died in 2019 or after, availability was lower for those who did not have TDP-43 present in the hippocampus (393 out of 469, 84%) compared to those who did (241 out of 256, 94%, Chi-square *P* < 0.001).

### Part II, complete case analysis: frequency of TDP-43 neuropathologies in NACC

After examining availability of TDP-43 measures in the NACC database in Part I, we then focused on the subset of participants who had all the relevant TDP-43 pathological assessments (TDP-43 assessed for all brain regions, FTLD-TDP, ALS-TDP, and HS-A) available to examine frequency of the TDP-43 groups in this subset of NACC data, distribution of regional TDP-43 inclusions, and associations with clinical diagnosis and other pathologies. All TDP-43 assessments were available for *N* = 2142 participants, from 31 ADCs, representing 50% of the 4326 participants with version 10 + pathology data. We show the flow diagram for the TDP-43 categories assigned in Fig. 1b. LATE-NC was the most prevalent TDP-43 pathology group (*N* = 580, 27%). ALS or FTLD-TDP (ALS/FTLD-TDP) was present in 185 participants (9%); of these participants, 146 (79%) had FTLD-TDP only, 29 (16%) had both ALS-TDP and FTLD-TDP, and 10 (5%) had ALS-TDP only. In addition to the participants with ALS-TDP, there were five other participants with non-TDP-43 ALS inclusions in motor neurons: one had FUS inclusions, one had SOD1 inclusions, and three were listed as non-specific inclusions. Lastly, there were 41 participants (2%) who had brain TDP-43 but did not have ALS/FTLD-TDP and had some exclusionary criteria for LATE-NC. Of these, 11 had CBD, 6 had CTE, 5 had acute TBI as assessed at autopsy, 3 had chronic TBI as assessed at autopsy, 6 had an FTD-related mutation, and 5 had ADAD mutations. There were also 9 participants who did not have pathology or genetic characteristics that would exclude them from being assigned LATE-NC, but instead had neocortical inclusions while missing TDP-43 in the amygdala and/or hippocampus: of these only 2 had TDP-43 only in the neocortex, the rest having TDP-43 inclusions in the other regions but not in both the amygdala and hippocampus. We mention center-specific findings for TDP-43 group frequencies in the Supplementary Text and display the distribution of TDP-43 groups in Supplementary Fig. 4a, where frequencies of the different TDP-43 groups varied widely across centers. We also show, within those classified as LATE-NC, the distribution of LATE-NC stages across centers in Supplementary Fig. 4b, where for most centers stage 2 was the most common. We show histograms with ages at death in Supplementary Fig. 5, which mostly followed expected trends: participants with LATE-NC tended to die at older ages, participants with ALS/FTLD-TDP tended to die at younger ages, and those in the Other TDP-43 group were a combination of younger and older ages at death.

### Part II, complete case analysis: distribution of regional TDP-43

**Fig. 4:**
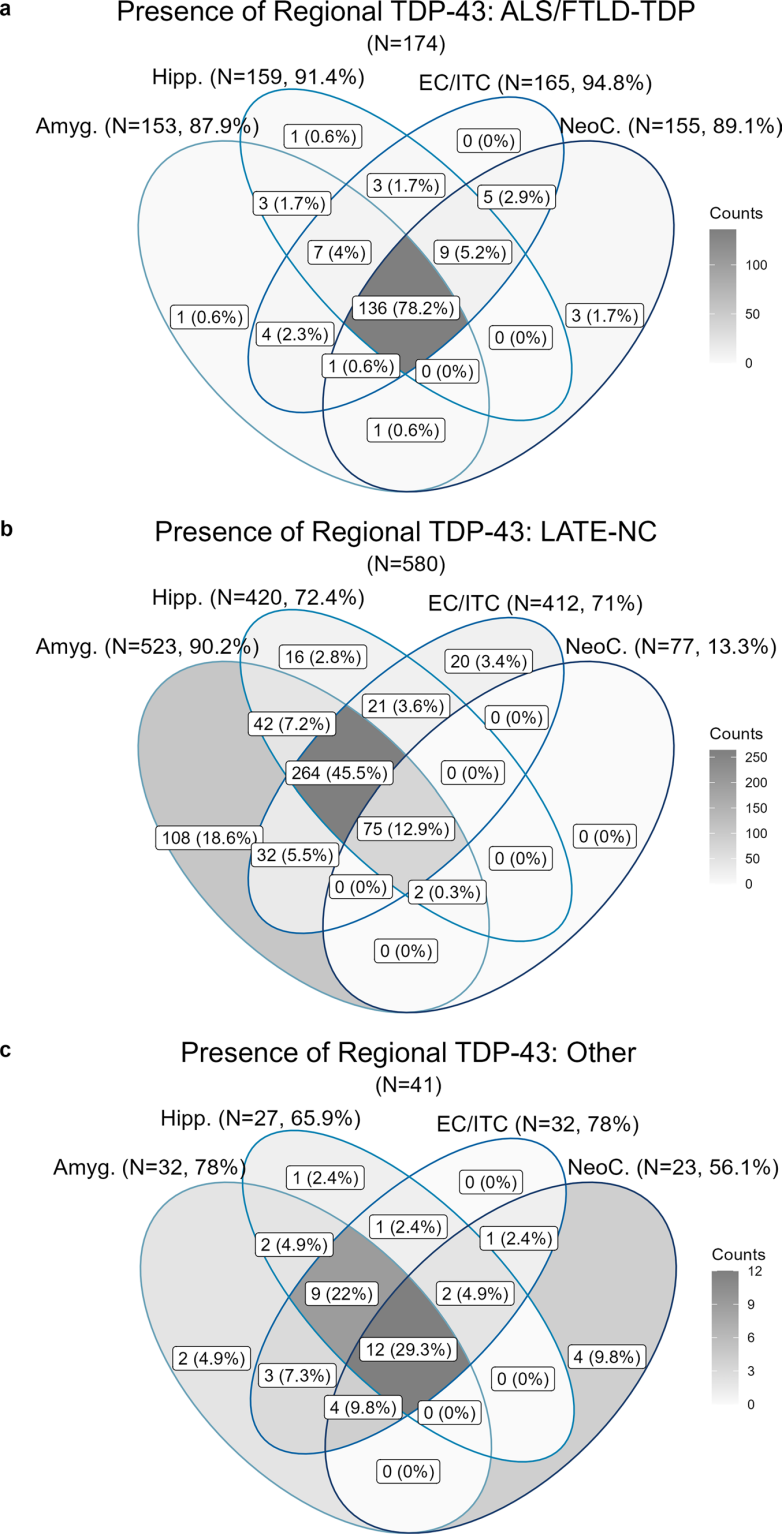
Venn diagrams for presence of regional TDP-43 inclusions by pathological category ALS/FTLD-TDP (**a**), LATE-NC (**b**), and Other TDP-43 (**c**). *ALS/FTLD-TDP* amyotrophic lateral sclerosis or frontotemporal lobar degeneration with TDP-43 pathology, *LATE-NC* limbic-predominant age-related TDP-43 encephalopathy neuropathologic change, *Amyg*. amygdala, *Hipp.* hippocampus, *EC/ITC* entorhinal cortex/inferior temporal cortex, *NeoC.* neocortex

We next examined the regional distribution of TDP-43 inclusions across the different TDP-43 groups. For ALS-TDP, 4 participants did not have brain regional TDP-43 but did have TDP-43 present in the spinal cord, while 1 participant with ALS-TDP and 6 participants with FTLD-TDP did not have any regional TDP-43 noted, potentially signaling a data entry error or the presence of TDP-43 inclusions in a region not included in the NACC neuropathology form. Figure 4 shows the patterns of regional TDP-43 inclusions (in those with TDP-43 present in at least one brain region) for those with ALS/FTLD-TDP, LATE-NC, or Other TDP-43. Participants with ALS/FTLD-TDP (Fig. 4a) generally had TDP-43 inclusions across all the assessed brain regions (78%), with the EC/ITC being the single most common region (95%) followed by the hippocampus (91%), neocortex (89%) and amygdala (88%). For LATE-NC, 34% were classified as stage 1 (isolated TDP-43 inclusions in amygdala, hippocampus, or EC/ITC), 53% were stage 2 (TDP-43 inclusions in both the amygdala and hippocampus), and 13% were stage 3 (TDP-43 inclusions in the amygdala, hippocampus, and neocortex). The distribution of TDP-43 inclusions in participants with LATE-NC (Fig. 4b) showed significant variation at early-stage regions. The most common pattern of LATE-NC TDP-43 inclusions was the typical stage 2 (TDP-43 inclusions in the amygdala, hippocampus, and EC/ITC, 46%), followed by the typical stage 1 (TDP-43 inclusions in the amygdala alone, 19%). Other stage 1 configurations were not entirely uncommon, with 6% made up of isolated hippocampal or EC/ITC (3% each) inclusions, and EC/ITC inclusions with hippocampal (4%) or amygdala (6%) inclusions. Also, it was relatively rare to see LATE-NC stages 2 (7%) and 3 (< 1%) without EC/ITC inclusions. Lastly, participants in the “Other TDP-43” category (Fig. 4c) were a combination of TDP-43 in all regions (29%), neocortical TDP-43 without inclusions in amygdala or hippocampus (27%) or some combination of temporal lobe regions (44%). Though less widely available, we briefly discuss findings with respect to spinal cord TDP-43 in the Supplementary Text.

### Part II, complete case analysis: associations of TDP-43 pathologies with clinical diagnoses and other neuropathologies

We examined the demographic, clinical, and co-occurring pathology characteristics of the cohort split into ALS/FTLD-TDP, LATE-NC, Other TDP-43, and those without brain TDP-43, which we display in Table 1. Participants with ALS/FTLD-TDP had on average the youngest age at death (mean 72 years), participants with LATE-NC had the highest average age at death (mean 84 years), the TDP-43 Other group also tended to be a bit older (mean 81 years), compared to those with No TDP-43 (mean 79 years). Participants with LATE-NC had a significantly higher frequency of APOE4 (58%) compared to the other groups (~ 30%). As expected, the ALS/FTLD-TDP group had the highest frequency of FTD-related mutations (23%), while LATE-NC, by definition, did not have any, and there was a relatively high frequency in the Other TDP-43 group (15%). Those with LATE-NC had the highest frequency of phospho-specific TDP-43 antibody used for TDP-43 assessment (79%).

**Fig. 5:**
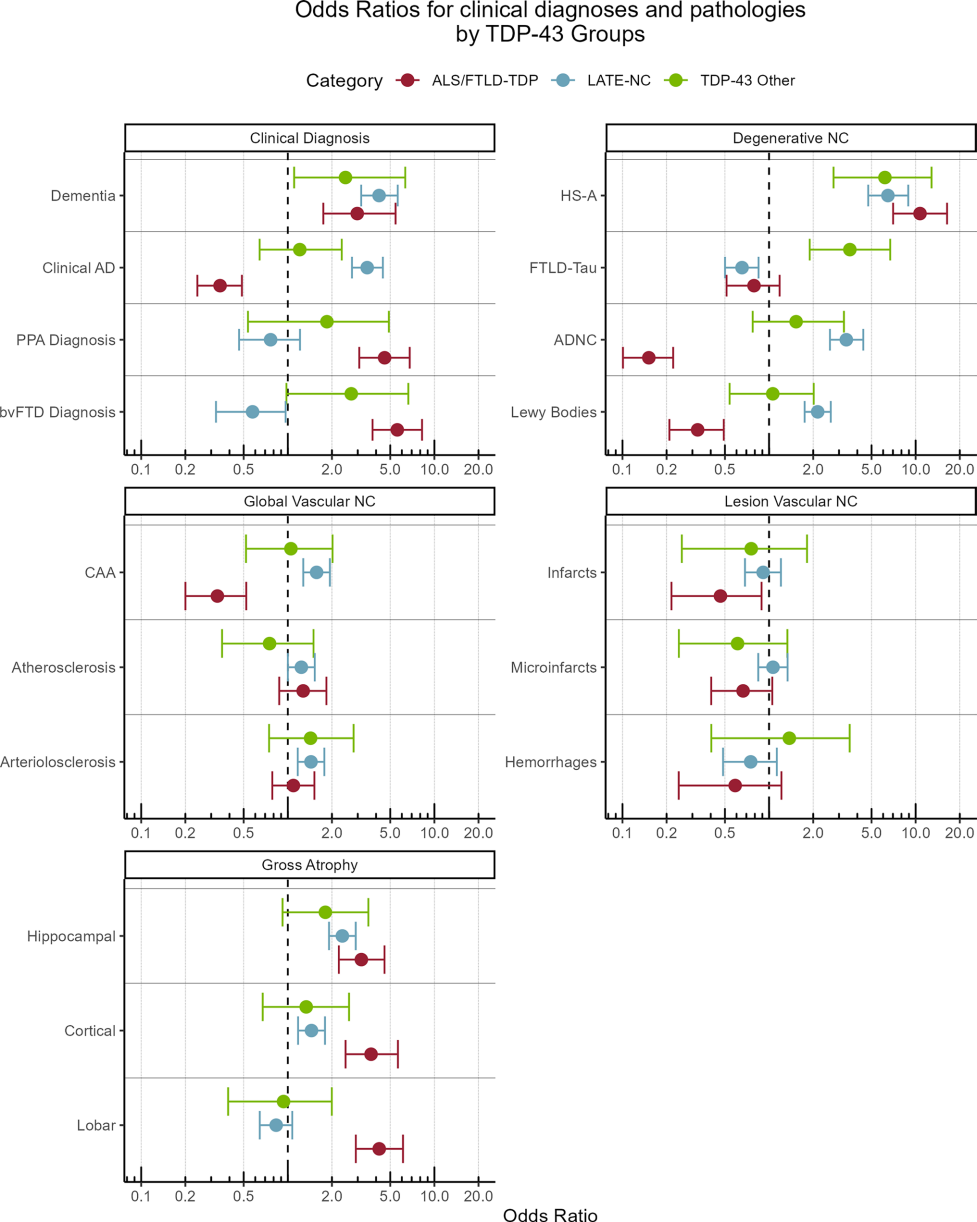
Odds ratios for various clinical diagnoses and other neuropathologic changes with regards to TDP-43 categories, ALS/FTLD-TDP, LATE-NC, and Other TDP-43, compared to those without TDP-43. Logistic regressions adjusted for age at death, sex, education, and interval between last visit and death. Dots represent odds ratios and error bars represent 95% confidence intervals. *LATE-NC* limbic-predominant age-related TDP-43 encephalopathy neuropathologic change, *ALS/FTLD-TDP* amyotrophic lateral sclerosis or frontotemporal lobar degeneration with TDP-43 pathology, *AD* Alzheimer’s disease, *PPA* primary progressive aphasia, *bvFTD* behavioral variant of frontotemporal dementia, *HS-A* hippocampal sclerosis of aging, *ADNC *Alzheimer’s disease neuropathologic change, *CAA* cerebral amyloid angiopathy

Results for the logistic regressions of clinical diagnoses and co-occurring pathologies by TDP-43 groups are shown in Table 2 and in Fig. 5, which we discuss next. With regards to clinical diagnoses, all TDP-43 groups were associated with increased odds of dementia compared to those without TDP-43 (LATE-NC OR = 4.2, ALS/FTLD-TDP OR = 3.0, Other TDP-43 OR = 2.5). While LATE-NC was associated with increased odds of clinical AD diagnosis (OR = 3.5), ALS/FTLD-TDP was associated with lower odds of a clinical AD diagnosis (OR = 0.3) but higher odds of PPA (OR = 4.6) and bvFTD (OR = 5.6) diagnosis. The Other TDP-43 group was also associated with increased odds of bvFTD (OR = 2.7) while LATE-NC was associated with lower odds (OR = 0.6).

**Table 2:** Results from logistic regressions for clinical diagnosis and co-pathologies by TDP-43 category: LATE-NC, ALS/FTLD-TDP, or Other TDP-43. Logistic regressions adjusted for age at death, sex, education, and interval between last visit and death. *P* values for post-hoc contrasts between the groups are shown on the right hand side of the table

Variable	Other TDP-43			LATE-NC			ALS/FTLD-TDP			Post-hoc tests		
	OR	95% C.I	*P* value	OR	95% C.I	*P* value	OR	95% C.I	*P* value	LATE vs. ALS/FTLD	LATE vs. Other	ALS/FTLD vs. Other
Clinical diagnosis												
Dementia	2.5	[1.1,6.3]	0.039	4.2	[3.2,5.6]	< 0.001	3.0	[1.7,5.4]	< 0.001	0.690	0.648	0.984
Clinical AD	1.2	[0.6,2.3]	0.564	3.5	[2.7,4.5]	< 0.001	0.3	[0.2,0.5]	< 0.001	< 0.001	0.010	0.003
PPA diagnosis	1.8	[0.5,4.9]	0.264	0.8	[0.5,1.2]	0.266	4.6	[3.1,6.8]	< 0.001	< 0.001	0.421	0.378
bvFTD diagnosis	2.7	[1,6.6]	0.039	0.6	[0.3,1]	0.046	5.6	[3.8,8.2]	< 0.001	< 0.001	0.019	0.474
Degenerative NC												
HS-A	6.2	[2.8,12.8]	< 0.001	6.5	[4.7,8.9]	< 0.001	10.7	[7,16.4]	< 0.001	0.068	> 0.999	0.530
FTLD-Tau	3.6	[1.9,6.7]	< 0.001	0.7	[0.5,0.8]	0.002	0.8	[0.5,1.2]	0.264	0.856	< 0.001	< 0.001
ADNC	1.5	[0.8,3.2]	0.240	3.4	[2.6,4.4]	< 0.001	0.2	[0.1,0.2]	< 0.001	< 0.001	0.155	< 0.001
Lewy bodies	1.1	[0.5,2]	0.861	2.1	[1.7,2.6]	< 0.001	0.3	[0.2,0.5]	< 0.001	< 0.001	0.162	0.014
Global vascular NC												
CAA	1.0	[0.5,2]	0.887	1.6	[1.3,1.9]	< 0.001	0.3	[0.2,0.5]	< 0.001	< 0.001	0.655	0.026
Atherosclerosis	0.8	[0.4,1.5]	0.430	1.2	[1,1.5]	0.049	1.3	[0.9,1.8]	0.201	0.999	0.528	0.553
Arteriolosclerosis	1.4	[0.7,2.8]	0.287	1.4	[1.2,1.8]	< 0.001	1.1	[0.8,1.5]	0.603	0.446	> 0.999	0.883
Lesion vascular NC												
Infarcts	0.8	[0.3,1.8]	0.568	0.9	[0.7,1.2]	0.518	0.5	[0.2,0.9]	0.032	0.267	0.982	0.851
Microinfarcts	0.6	[0.2,1.3]	0.248	1.1	[0.8,1.3]	0.598	0.7	[0.4,1.1]	0.094	0.256	0.573	0.998
Hemorrhages	1.4	[0.4,3.5]	0.556	0.7	[0.5,1.1]	0.180	0.6	[0.2,1.2]	0.189	0.945	0.700	0.570
Gross atrophy												
Hippocampal	1.8	[0.9,3.5]	0.084	2.4	[1.9,2.9]	< 0.001	3.2	[2.2,4.6]	< 0.001	0.428	0.869	0.443
Cortical	1.3	[0.7,2.6]	0.400	1.5	[1.2,1.8]	< 0.001	3.7	[2.5,5.6]	< 0.001	< 0.001	0.995	0.048
Lobar	0.9	[0.4,2]	0.871	0.8	[0.6,1.1]	0.161	4.2	[2.9,6.1]	< 0.001	< 0.001	0.993	0.004

*LATE-NC* limbic-predominant age-related TDP-43 encephalopathy neuropathologic change, *ALS/FLTD-TDP*: amyotrophic lateral sclerosis or frontotemporal lobar degeneration with TDP-43, *OR* odds ratio, *CI* confidence interval, *AD* Alzheimer’s disease, *PPA* primary progressive aphasia, *bvFTD* behavioral variant of frontotemporal dementia, *HS-A* hippocampal sclerosis of aging, *ADNC* Alzheimer’s disease neuropathologic change, *CAA* cerebral amyloid angiopathy

**Fig. 6:**
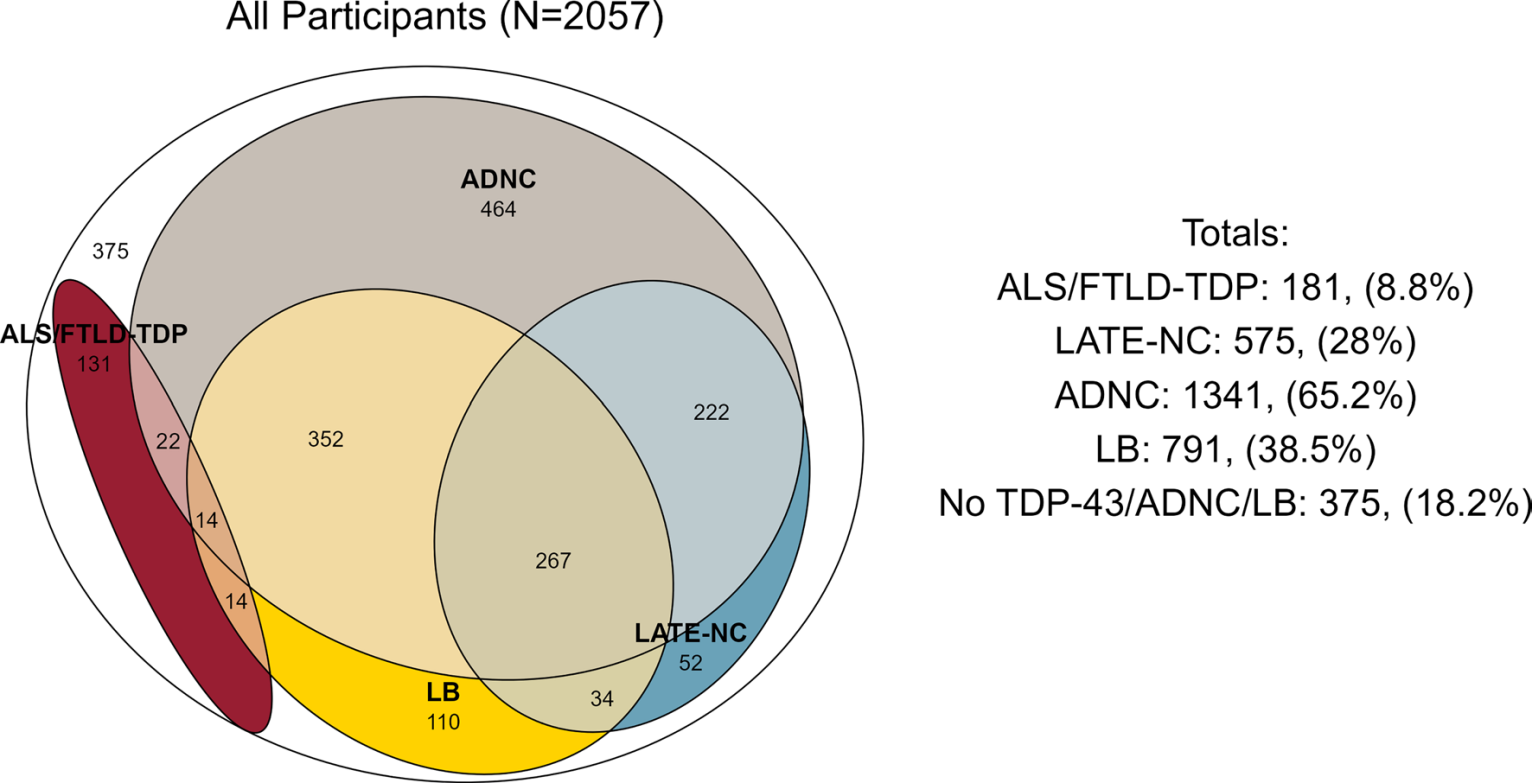
Proportional Euler diagrams depicting co-occurrence of ALS/FTLD-TDP, LATE-NC, Alzheimer’s disease neuropathologic change (ADNC), and Lewy bodies (LB), in overall cohort excluding participants with “Other TDP-43” or without ADNC or LB ratings (*N* = 2057). *ALS/FTLD-TDP* amyotrophic lateral sclerosis or frontotemporal lobar degeneration with TDP-43 pathology, *LATE-NC* limbic-predominant age-related TDP-43 encephalopathy neuropathologic change, *ADNC* Alzheimer’s disease neuropathologic change, *LB* Lewy bodies

With regards to other degenerative pathologies, all the TDP-43 groups were strongly associated with increased odds of HS-A (LATE-NC OR = 6.5, ALS/FTLD-TDP OR = 10.5, Other TDP-43 OR = 6.2). LATE-NC was associated with decreased odds of FTLD-tau (OR = 0.7) while Other TDP-43 was associated with increased odds (OR = 3.6). LATE-NC was associated with higher odds for ADNC (OR = 3.4) and LB (OR = 2.1), while the opposite was the case for ALS/FTLD-TDP (ADNC OR = 0.2, LB OR = 0.3). With regards to global vascular pathologies, LATE-NC was associated with increased, and ALS/FTLD-TDP decreased, odds of CAA, but these findings were weaker than those for ADNC. LATE-NC was weakly but significantly associated with increased odds of atherosclerosis (OR = 1.2) and arteriolosclerosis (OR = 1.4). For lesion vascular pathologies, those with ALS/FTLD-TDP had lower odds of gross infarcts (OR = 0.5) and a trend for lower odds of microinfarcts (OR = 0.7, *P* = 0.09). For gross atrophy measures, ALS/FTD-TDP (OR = 3.2) and LATE-NC (OR = 2.4) were significantly associated with, and Other TDP-43 (OR = 1.8, *P* = 0.084) trended for, increased odds of hippocampal atrophy. LATE-NC (OR = 1.5) and ALS/FTLD-TDP (OR = 3.7) were significantly associated with increased odds of cortical atrophy, but only ALS/FTLD-TDP was significantly associated with increased odds of frontal/temporal lobar atrophy. For illustrative purposes, we show a proportional Venn diagram in Fig. 6 with the overlap between a few pathologies: ALS/FTLD-TDP, LATE-NC, ADNC, and LB, excluding those with “Other TDP-43” or missing assessments for ADNC or LB. In this figure we see a high degree of overlap of LATE-NC with ADNC and LBs, but much less overlap between ALS/FTLD-TDP, ADNC, and LBs. Results for associations with clinical diagnoses and other pathologies were largely similar for the multilevel models accounting for center, which we show in Supplementary Table 1 and Supplementary Fig. 6.

We also examined differences in regional TDP-43 and participant characteristics for those with both ALS-TDP and FTLD-TDP compared to those with either ALS-TDP or FTLD-TDP by itself. Those with both ALS-TDP and FTLD-TDP tended to have the most widespread inclusions (98% had TDP-43 in all regions), those with FTLD-TDP alone also tended to have extensive TDP-43 inclusions (77% TDP-43 in all regions), while those with ALS-TDP alone had more isolated TDP-43 (Supplementary Fig. 7). Those with both ALS-TDP and FTLD-TDP tended to die younger, were more likely to have bvFTD, and those with FTLD-TDP only had the highest rates of HS-A (Supplementary Table 2). We further describe these findings in the Supplementary Text.

## Discussion

### Summary

In this study we documented the availability of TDP-43 pathology data in the NACC neuropathology database and report findings (including some novel results) using the subset of participants with all TDP-43 related pathology information available. We found that participants with clinical FTLD had higher follow-up, autopsy, and TDP-43 assessment rates, compared to participants with clinical AD. We found that HS-A and ALS assessments were available for most participants, while FTLD-TDP assessment was less widely available, and regional TDP-43 assessments (amygdala, hippocampus, EC/ITC, and neocortex) were even less available and varied in their availability by the respective contributing ADCs. However, the availability of most TDP-43 related pathologies has increased over time, with a high degree of availability for these measures from participants who died in 2022 with most measures available in ~ 90% of participants.

We defined ALS/FTLD-TDP as those with either ALS-TDP or FTLD-TDP presence. We used a category of “Other TDP-43” to capture participants with TDP-43 in their brains who were not assigned as ALS/FTLD-TDP but showed genetic or pathology characteristics which would rule out LATE-NC. We defined LATE-NC as the remaining participants with brain TDP-43 pathology. In the sample of participants with all TDP-43 information available, we found that 27% had LATE-NC, 9% of participants had ALS-TDP or FTLD-TDP, and 2% had Other TDP-43, while 62% did not have brain TDP-43 pathology detected. Participants with ALS/ FTLD-TDP generally had TDP-43 in all regions, and we found high rates of TDP-43 in temporal lobe regions. In LATE-NC, there was variation in the distribution of TDP-43 inclusions across the amygdala, hippocampus, and EC/ITC. With regards to clinical diagnoses and other co-occurring pathologies, all TDP-43 categories (LATE-NC, ALS/FTLD-TDP, Other TDP-43) had higher odds of dementia and presence of HS-A compared to those without TDP-43. LATE-NC was associated with a higher frequency of clinical AD diagnosis, ADNC, Lewy bodies, atherosclerosis, arteriolosclerosis, and hippocampal and cortical atrophy, while ALS/FTLD-TDP was associated with increased odds of PPA and bvFTD clinical diagnoses as well as cortical and frontal/temporal lobar atrophy, but lower odds of a clinical diagnosis of clinical AD, ADNC, and LB. The “Other TDP-43” group seemed to share some features with the LATE-NC and ALS/FTLD-TDP groups but did have higher odds of FTLD-tau and bvFTD compared to those without TDP-43.

### Part I findings

The NACC database is not representative of the general population in key ways such as older age and higher levels of education of participants, a lack of racial and ethnic diversity, and interactions between these factors [6]. A recent study documented some of the center-specific trends with respect to recruitment, finding differences between centers with regards to the presumptive etiologic diagnosis at first visit in relation to age, APOE4 status, and high frequency of rare (i.e. FTD) and vascular pathologies [25]. Our study complements these findings by examining the last available presumptive clinical etiologic diagnosis, with a focus on the most recent NACC neuropathology forms. We found that participants with clinical FTLD at last visit were more likely to be followed until death, have autopsy assessment, and have assessment for TDP-43. These results are expected, and the enrichment for rarer neuropathologies actually allows for comparisons between groups as we have performed in this study. However, it should be underscored that the frequency of these pathologies found in this study is different from that in the general population.

The increase in TDP-43 assessment availability over time are encouraging signs and signal that the value of the NACC database for studying TDP-43 related neuropathologies is growing. Despite these more recent trends in increased availability, the retrospective data is still most limited by the FTLD-TDP and regional TDP-43 assessments. While regional TDP-43 assessments have increased in availability by year, there was considerable center-to-center variability in the anatomic regions assessed for TDP-43 pathology. For example, some centers performed TDP-43 immunohistochemical stains, but only reported whether FTLD-TDP or ALS-TDP were present but did not record any regional TDP-43 measures, signaling that they may have been more focused on etiology rather than distribution of pathology. While centers tended to assess TDP-43 in each region at similar rates overall, some centers sampled some regions less frequently than the others. In particular, while hippocampal assessment was widespread, the amygdala, EC/ITC, or neocortical tended to be sampled less consistently. It has also been shown that different ADCs employ different TDP-43/pTDP-43 antibodies [42]. Even given these differences in methodologies, these research centers represent the “state of the art” of neuropathologic workups in U.S. ADCs. We also observed systematic effects in TDP-43 sampling, as participants with TDP-43 found in the hippocampus were more commonly assessed for neocortical TDP-43 beginning in 2019 than those without hippocampal TDP-43. This supports the notion that the original LATE-NC paper [62] catalyzed a change in neuropathologic practices overall. However, when we examined participants with all regional assessments available there was still a small subset of participants with neocortical TDP-43 without hippocampal TDP-43, both in those with and those without ALS/FTLD-TDP. It is possible that some of these cases may have CTE that was not detected, since this finding of TDP-43 inclusions in the cortex but not the hippocampus has been shown in CTE-related cases [66]. Nonetheless, this finding of isolated neocortical TDP-43 may be worth investigating further.

Based on our findings from Part I, we recommend other researchers carefully consider the incomplete availability of TDP-43 assessments in NACC and try to identify ways to maximize the available information. The change in availability in neocortical TDP-43 after 2019 is a case in point for motivating researchers to devise specific inclusion criteria depending on the purpose of the study. Additionally, the hippocampal TDP-43 assessment may be useful as a single region for analyses concerning LATE-NC as it is strongly related to LATE-NC stage 2 or higher and was the most widely available regional TDP-43 assessment.

### Part II findings

In NACC participants with all TDP-43 pathology information available, around 30% of participants had LATE-NC and around 10% had ALS/FTLD-TDP. This is in contrast to estimates of the prevalence of FTD in the general population of around 10 cases per 100,000 (0.01%) [22, 43]. However, a recent large multisite study examining 13 community cohorts totaling over 6000 individuals found that LATE-NC was present in around 40% of participants and FTLD-TDP was found in none of these [61]. While the high percentage of ALS/FTLD-TDP participants in NACC is not reflective of the general population, it does pool a large number of participants and enables examining these rare pathological entities, including comparisons with LATE-NC. The “Other TDP-43” group, serving primarily to rule out participants from the LATE-NC group, was a combination of FTLD-tau pathologies (CBD and CTE), acute or chronic TBI as assessed at autopsy, and participants with FTD-related or ADAD-related mutations. We did not find other rare conditions such as Huntington disease and Parkinsonism of Guam and Kii in the subset of participants we analyzed. Overall, the “Other TD-43” category represented about 2% of participants with all TDP-43 assessments available, and no single condition seemed to be dominant. For example, while CTE has a very strong association with TDP-43 [66], with only 6 participants with CTE in the “TDP-43 Other” group this may be a minor factor with respect to TDP-43 pathologies in NACC. Additionally, some of the participants in the “Other TDP-43” group may have been mis-classified. For example, those with FTD-related mutations but not designated as ALS/FTLD-TDP (*N* = 6) may represent such a misclassification, though some of these FTD-related mutations may have been related to tau and not TDP-43. Thus, these “Other TDP-43” factors may be most useful in excluding participants from the designation of LATE-NC, while the main TDP-43 categorizations present in the NACC neuropathology database do appear to be LATE-NC and ALS/FTLD-TDP. Based on our findings in Part II, we would recommend researchers carefully consider the high frequency of clinical FTLD and FTLD-TDP pathology in NACC, especially in the neuropathology dataset, and account for this through the use of neuropathology variables, clinical diagnoses, or both. While rarer, researchers should also account for the presence of other TDP-43 pathologies beyond ALS/FTLD-TDP or LATE-NC in NACC data as these were nonetheless still present.

Previous studies using NACC data have provided limited information regarding regional distribution of TDP-43, instead usually focusing on using these measures to define the presence of TDP-43 across any of these regions to assign pathological categories such as LATE-NC. Some previous studies evaluated associations with individual TDP-43 regions [42] and reported regional TDP-43 results for participants clustered by demographic characteristics and clinical diagnoses [41] but did not assess the co-occurrence of TDP-43 across these regions. By restricting the sample analyzed to those with all TDP-43 related pathology information available, we were able to examine not only the frequency of ALS-TDP, FTLD-TDP, and LATE-NC in NACC more precisely, as missing regional assessments can result in some degree of misclassification, but we were also able to examine the regional patterns of TDP-43 deposition across these different pathological conditions.

There was a subset of participants with ALS-TDP and no brain TDP-43 pathology, but most of these had TDP-43 pathology in the spinal cord. However, in this ADC-based cohort, most participants with ALS-TDP had TDP-43 inclusions in the brain. These results are in contrast with other studies that assessed regional distributions of TDP-43 in participants with ALS and found that while TDP-43 inclusions in the medial temporal lobe were relatively common, they were not nearly as prevalent as we found in this study [58, 67]. Additionally, a proposed staging criteria for ALS-TDP listed the hippocampus as the final stage [16]. However, these previous studies selected participants with ALS-TDP based on both clinical and pathology factors and thus likely represent a purer ALS-TDP phenotype. By contrast, in this study we defined ALS-TDP as a purely pathological category and saw a high overlap with FTLD-TDP, suggesting that the TDP-43 patterns reported here may be less reflective of traditional ALS and more reflective of FTLD-TDP with some ALS involvement. However, some previous studies in participants with clinical ALS have also found hippocampal atrophy on imaging [20, 50], which might suggest more prominent involvement of TDP-43 in this region in ALS, similar to what we have reported in this study.

In participants with ALS/FTLD-TDP, we found that widespread TDP-43 inclusions in all of the brain regions was common (~ 80%). This was true in the subset with FTLD-TDP only, but even more so in the subset with both ALS-TDP and FTLD-TDP, while the ALS-TDP only group often did not have brain TDP-43 and we found no consistent pattern in those who did. A previous analysis of sequential deposition of TDP-43 in FTLD-TDP, though noting variability in spread, suggested that deposition can begin in the amygdala and/or orbitofrontal cortex and then proceed to the hippocampus and middle frontal gyrus [15]. Also, criteria for FTLD-TDP subtypes rely exclusively on the distribution and types of neocortical TDP-43 inclusions [52, 53, 64]. However, in our current analysis, the most common regions with TDP-43 were the EC/ITC and the hippocampus, though again most regions had TDP-43 most of the time. In agreement with our results, a previous study also found hippocampal TDP-43 in a high percentage of participants with FTLD-TDP (80%) [71], which, while slightly lower than our percentage, does concur with our results and together suggest a more prominent role for medial temporal TDP-43 in participants with FTLD-TDP.

As expected, participants with LATE-NC were significantly older, and participants with ALS/FTLD-TDP significantly younger, at death than those with without TDP-43. Previous studies examining the age distribution of participants with ALS-TDP [58] and FTLD-TDP [18] defined their upper age group (i.e. “older”) as those over the age of 75. These previous studies, however, selected participants not only using neuropathological criteria but also clinical or genetic information, whereas our designation was based on neuropathological criteria alone. We found a subset of participants in NACC who were classified as having ALS/FTLD-TDP but died at oldest-old ages (over 85). This may be due to potential misclassification of LATE-NC as FTLD-TDP in cases with particularly severe TDP-43 deposition, such as the discrepancies between pathologist ratings for LATE-NC and FTLD-TDP for selected cases previously described [68]. Additionally, in the aforementioned age-based examinations of ALS-TDP [58] and genetically-confirmed FTLD-TDP [18] the authors found that in older ALS/FTLD-TDP participants the distribution of TDP-43 was more heavily limbic and thus resembling LATE-NC more than when found at younger ages. The LATE-NC group had the highest rate of phospho-specific TDP-43 antibody use, indicating that the phospho-specific antibody may be more sensitive to the potentially subtle TDP-43 inclusions in LATE-NC and thus LATE-NC was detected more often when this test was used, whereas this was not the case for ALS/FTLD-TDP where TDP-43 inclusions are usually more widespread.

With regards to cognitive symptoms and diagnoses, LATE-NC was associated with dementia and a clinical diagnosis of AD, in line with previous reports [13, 38, 56, 62, 79]. Participants with ALS/FTLD-TDP, while also having higher odds of dementia, had higher odds of PPA and bvFTD but lower odds of a clinical diagnosis of AD. These findings recapitulate a previous study using NACC data [74] and are also in agreement with a study from the Mayo Clinic which also contrasted various TDP-43 related neuropathological entities including FLTD-TDP, LATE-NC with ADNC, and LATE-NC without ADNC [17]. While focused on brain atrophy patterns, this previous study from the Mayo clinic also compared the clinical characteristics of those with FTLD-TDP and those with LATE-NC and found a higher frequency of bvFTD and PPA in those with FTLD-TDP and a higher frequency of clinical AD in those with LATE-NC.

In this present study LATE-NC was significantly associated with HS-A, ADNC, LB, CAA, atherosclerosis and arteriolosclerosis. Participants with ALS/FTLD-TDP, on the other hand, had greater odds of HS-A but lower odds of ADNC, LB, and CAA, compared to NACC participants without brain TDP-43. The association between TDP-43 and HS-A, is well known for both FTLD-TDP [7, 31, 71] and LATE-NC [4, 32, 60, 72]. A novel contribution from this study, however, is the similarities in odds of HS-A for ALS/FTLD-TDP, LATE-NC, and even in the “Other TDP-43” group, compared to those without TDP-43. While previous studies have emphasized differences in some of the characteristics of ALS/FTLD-TDP-related and LATE-NC-related HS-A, namely worse global brain atrophy and synaptic loss in CA1 in those with ALS/FTLD-TDP compared to LATE-NC, nonetheless HS-A across these two groups shared similarities such as the levels of neuronal loss and hippocampal atrophy [3]. The co-occurrence of LATE-NC and ADNC has also been reported previously [36, 37], while the negative association between ALS/FTLD-TDP and ADNC may have to do with the higher frequency of ADNC in NACC as a whole and the fact that participants with ALS/FTLD-TDP are often recruited due to their specific cognitive problems or family history leading to a higher likelihood of having a non-AD underlying pathology. LATE-NC was also associated with LB in line with previous reports [2]. Indeed, LB, ADNC, and LATE-NC seem to co-occur, as we have found in this study, and this combination has been shown to be particularly detrimental for cognitive function [39]. While vascular pathologies were not as strongly related to the TDP-43 pathologies, we did find statistically significant associations for LATE-NC with CAA, atherosclerosis, and arteriolosclerosis.

The results for gross assessment of atrophy also followed expected trends, with all of LATE-NC, ALS/FTLD-TDP, and Other TDP-43, being related to hippocampal atrophy. Furthermore, while both LATE-NC and ALS/FTLD-TDP were also associated with cortical atrophy, the effect was much stronger for ALS/FTLD-TDP. This is in line with previous literature on LATE-NC where atrophy of limbic structures is present early on [47], but cortical atrophy is only associated with the latter stages of LATE-NC [10]. The hippocampus is also a common site for neurodegeneration across various neuropathologic changes [82]. We found ALS/FTLD-TDP was also associated with frontal/temporal lobar atrophy, which is well documented in the literature albeit with variations in atrophy patterns amongst FTLD-TDP subtypes [14, 69, 78]. While few studies have directly compared atrophy patterns between ALS/FTLD-TDP and LATE-NC, our findings concur with a recent study which found higher rates of both cortical and hippocampal atrophy (from MRI) in FTLD-TDP compared to LATE-NC [17]. These assessments of gross atrophy at autopsy provide important insights into the expected in vivo atrophy patterns, which is especially pertinent to LATE-NC given a dearth of neuroimaging studies in the oldest-old [81].

In this study we found a high overlap of ALS-TDP and FTLD-TDP, which concurs with the idea that ALS-TDP and FTLD-TDP are often on the same spectrum [19, 28, 49]. Participants with both ALS-TDP and FTLD-TDP tended to die at younger ages than those with either pathology alone. Those with ALS-TDP only tended to have less severe cognitive impairment. A previous study comparing the different combinations of ALS-TDP and FTLD-TDP did not find significant differences in age at death but did find a higher prevalence of cognitive impairment in those with FTLD-TDP [73]; however, this study selected participants based on both clinical and pathological criteria. In our results, those with FTLD-TDP only had the highest prevalence of PPA, HS-A, and hippocampal, cortical, and lobar atrophy. This finding likely relates to differences in FTLD-TDP subtypes, where type A (which is the most common) is not associated with ALS but is associated with worse hippocampal cell loss and frontotemporal atrophy while type B is associated with ALS symptoms and more limited cortical atrophy [51, 64, 69, 78]. Thus, while lacking information on FTLD-TDP subtypes in NACC, we seem to have found some FTLD subtype-related differences by accounting for ALS-TDP.

Our results for the diverging patterns of associations for ALS/FTLD-TDP and LATE-NC in relation to participant characteristics, clinical diagnoses, and co-occurring pathologies, are largely in agreement with a previous study using NACC data where they included participants with TDP-43 presence and identified different clusters of participants using measures of global cognition, PPA, bvFTD, and neuropsychological assessment, along with age at death [41]. They found one cluster comprised participants who tended to die younger, had a higher rate of FTLD-TDP pathological designation, more symptoms of PPA and bvFTD, and lower ADNC burden, while other clusters involved participants who died older, less often with FTD diagnosis, and generally less widespread TDP-43. The authors argued that these clusters represent FTLD-TDP and different variations of LATE-NC. Our study differs from this previous study on a few key points. First, we defined FTLD-TDP and LATE-NC based on the neuropathology designation, instead of taking TDP-43 positive participants and clustering them by clinical manifestations and age at death. Second, we used information from participants with all TDP-43 information available which we also then carefully examined. Third, we examined associations with symptoms and co-occurring pathologies for LATE-NC and ALS/FTLD-TDP, compared to those without TDP-43 instead of amongst those with TDP-43. And finally, we report on a few additional key neuropathologic measures such as gross cortical and frontal/temporal lobar atrophy.

### Limitations

There are a few limitations pertinent to the NACC dataset. It is not representative of the general population and instead reflects the recruitment schemes of the respective contributing ADCs. This is exemplified in the high frequency of rare pathologies, such as FTLD-TDP and ALS-TDP. Because we aimed to be comprehensive in our analyses, our reference group included all participants that did not have TDP-43, and as such this reference group included a diagnostic variety of participants with other rare pathologies such as forms of FTLD-tau, amongst others. Additionally, because we limited our analyses to those with all TDP-43 information available, this represents a “complete case” analysis which used about half of the participants with version 10 + neuropathology data. This enabled us to systematically assign TDP-43 pathology groups and to contrast actual measured regional distribution of TDP-43 between these groups. However, future studies may leverage patterns of availability and TDP-43 deposition for assigning TDP-43 categories and LATE-NC stages. Furthermore, because of the large number of potential ancillary variables, and a degree of redundancy in, for example, the TDP-43 regional assessments (i.e. hippocampal and EC/ITC regions are very closely related), NACC neuropathology data may be well suited for multiple imputation techniques for handling some of these missing data [45]. Another limitation concerns the ambiguity regarding the exact anatomical region sampled for some of the regional TDP-43 assessments. Specifically, there could be considerable variability in the EC/ITC due to ambiguity of which region (EC or ITC) is assessed, and in the “neocortical” TDP-43 assessment depending on whether the middle frontal gyrus (relevant to LATE-NC and FTLD-TDP) or the motor cortex (more relevant to ALS-TDP) were sampled. Furthermore, the lack of spinal cord TDP-43 assessments limits the utility of this dataset for analyses more focused on this region or on ALS-TDP. More refined pathological designations, such as FTLD-TDP subtypes or different types of TDP-43 inclusions, are also not part of the NACC neuropathology forms and may have provided more clarity for some of the results in the ALS/FTLD-TDP participants. However, because the purpose of NACC is aggregating data from many centers to increase numbers and facilitate collaboration across ADCs, standardized and more easily implementable neuropathology protocols are more important than more fine-grained approaches, and here we have leveraged these increased numbers to provide novel insights into TDP-43 pathologies.

## Conclusion

TDP-43 related neuropathologies such as ALS/FTLD-TDP, LATE-NC, and HS-A, are important contributors to dementia, and the NACC database is an important resource for studying these pathologies.

### Supplementary Information

Below is the link to the electronic supplementary material.

Supplementary file1 (PDF 4536 KB)

## Data Availability

NACC data are publicly available upon request.
