# Supplementary material for: Comprehensive assessment of TDP-43 neuropathology data in the National Alzheimer’s Coordinating Center database
Source: Acta Neuropathol. 2024 Jun 19;147(1):103. doi: 10.1007/s00401-024-02728-8 (PMC11186885; doi:10.1007/s00401-024-02728-8)

Supplementary Materials for: Comprehensive Assessment of TDP-43 Neuropathology Data in the National Alzheimer's Coordinating Center Database

Davis C. Woodworth, PhD[a,b], Katelynn M. Nguyen, BS[a,b], Lorena Sordo, PhD[b,c], Kiana A. Scambray, MS[a,b], Elizabeth Head, PhD[b,c], Claudia H. Kawas, MD[a,b,d], María M. Corrada, ScD[a,b,e], Peter T. Nelson, MD, PhD[f], S. Ahmad Sajjadi, MD, PhD[a-c]

[a]Department of Neurology, University of California, Irvine, CA, USA

[b]Institute for Memory Impairments and Neurological Disorders, University of California, Irvine, CA, USA

[c]Department of Pathology and Laboratory Medicine, University of California, Irvine, CA, USA

[d]Department of Neurobiology and Behavior, University of California, Irvine, CA, USA

[e]Department of Epidemiology, University of California, Irvine, CA, USA

[f]Department of Pathology and Laboratory Medicine, University of Kentucky, Lexington, KY, USA

SUPPLEMENTARY TEXT

RESULTS

Part I, Availability: ADC-specific findings for TDP-43 measure availability and presence

We examined the availability of TDP-43 related pathology information across the 35 ADCs that contributed v10+ neuropathology data (**Supplementary Figure 3a)**. While most centers had almost complete records of ALS and HS-A, the overall availability and sampling schemes for regional TDP-43 and FTLD-TDP assessments were more varied. Notably, some sites showed a predilection towards particular sampling schemes for regional TDP-43. For example, the highest contributing site (N=380 total participants) had high availability of amygdala (98%) and hippocampus (99%) regional TDP-43 but considerably less so for EC/ITC (69%) and neocortical TDP-43 (54%). The second highest contributing site (N=286) had 100% assessment for hippocampus, 99% for neocortical, 95% for EC/ITC, but only 88% for amygdala. For the third highest contributing site (N=277), there was 100% assessment for TDP-43 in the hippocampus and neocortex but 46% for the amygdala and only 4% for the ETC/ITC. The fourth highest contributing site (N=275) had much lower rates of regional TDP-43 overall with ~30% for the amygdala, hippocampus, and EC/ITC and only 18% for the neocortex. These four highest contributing sites illustrate the heterogeneity in regional TDP-43 sampling across ADCs.

We display the percentage presence of TDP-43 pathology measures (within those with assessment available) for each of the contributing ADCs in **Supplementary Figure 3b**. Across centers, few participants had ALS-TDP, occurring at most in 6% of participants for a particular ADC, with many centers reporting no cases. For FTLD-TDP, while there were 13 ADCs reporting no cases, a small percentage of participants with FTLD-TDP was fairly common across sites, with 6 sites reporting 9% or more participants with FTLD-TDP, the highest having a rate of 21%. Regional brain TDP-43 presence varied by site but was around 20-40% across ADCs for the amygdala, hippocampus and EC/ITC, with a drop-off for neocortical TDP-43 positivity hovering around 10%. The site with most prevalent regional TDP-43 reported 45% for the amygdala and 18% for the neocortex.

Part II, Complete Case Analysis: Focus on ALS-TDP and FTLD-TDP

When examining spinal cord TDP-43 in those with the assessment available (N=911), 31 out of 32 (97%) participants with ALS-TDP had TDP-43 in the spinal cord, 41out of 100 (41%) participants with FTLD-TDP had

spinal cord inclusions including 18 of 77 (23%) of those without also having an ALS-TDP designation. For LATE-NC, 5 out of 185 (3%) participants had TDP-43 in the spinal cord, four were stage 2 or lower and only one was stage 3. In those with Other TDP-43, 3 out of 14 (21%) had spinal cord inclusions, and 8 of 602 (1%) participants without brain TDP-43 or ALS/FTLD-TDP classification had inclusions in the spinal cord.

We further examined the ALS/FTLD-TDP group by splitting it into those with FTLD-TDP only, those with ALS-TDP only, and those with both ALS-TDP and FTLD-TDP present. We show the distribution of brain TDP-43 inclusions across these categories in **Supplementary Fig. 7**. There were few participants with ALS-TDP alone (N=10) and only half of these had any brain TDP-43 (N=5) with a varied distribution mostly along hippocampal, EC/ITC, and neocortical regions (**Supplementary Fig. 7a**). Those with FTLD-TDP alone (**Supplementary Fig. 7b**) generally had TDP-43 in all regions assessed (77%), with most other cases occupying some of the adjacent middle regions in the Venn diagram, with EC/ITC being the most common region (96%) followed by the hippocampus (92%), neocortex and amygdala (88% each). Those with both FTLD-TDP and ALS-TDP (**Supplementary Fig. 7c**) had the highest frequency of TDP-43 in all regions (93%), and all participants had neocortical deposition. In those with both ALS-TDP and FTLD-TDP, only two cases did not have TDP-43 in all regions: an individual with TDP-43 in all regions except the amygdala, and an individual with isolated TDP-43 in the neocortex.

We show the group characteristics in **Supplementary Table 2**. Participants with both ALS-TDP and FTLD-TDP tended to die at a younger age (65 vs 72 for the other groups), and participants with ALS-TDP only were less likely to have dementia (20% vs >90% for the other groups). While groups were somewhat similar in terms of PPA frequency, with FTLD-TDP only being the highest (36%, ALS-TDP only was 22% and both was 18%), bvFTD was not present in the ALS-TDP only group, was present in 36% of the FTLD-TDP only group, but was the highest in the group with both (76%). For the co-occurring pathologies, HS-A was significantly more common in the FTLD-TDP only group (37%) compared to both the ALS-TDP only group (0%) and the combined ALS-TDP and FTLD-TDP group (10%). ADNC was significantly more common in the ALS-TDP only group

(38%) compared to both the FTLD-TDP only group (22%) and both group (3%). Lastly, the FTLD-TDP only group had the highest rate of hippocampal, cortical, and lobar atrophy.

SUPPLEMENTARY TABLES

Supplementary Table 1. Results from multilevel logistic regressions for clinical diagnosis and co-pathologies by TDP-43 category: LATE-NC, ALS/FTLD-TDP, or Other TDP-43. Logistic regressions with varying intercept by center, adjusted for age at death, sex, education, and interval between last visit and death. P-values for posthoc contrasts between the groups are shown on the right hand side of the table. Abbreviations: LATE-NC: limbic-predominant age-related TDP-43 encephalopathy neuropathologic change. ALS/FLTD-TDP: amyotrophic lateral sclerosis or frontotemporal lobar degeneration with TDP-43. OR: odds ratio. C.I.: confidence interval. MCI: mild cognitive impairment. CDR-SB: Clinical Dementia Rating sum-of-the-boxes. AD: Alzheimer's disease. PPA: primary progressive aphasia. bvFTD: behavioral variant of frontotemporal dementia. HS-A: hippocampal sclerosis of aging. ADNC: Alzheimer's disease neuropathologic change. CAA: cerebral amyloid angiopathy.

	Other TDP-43			LATE-NC			ALS/FTLD-TDP			Posthoc Tests		
Variable	OR	95% C.I.	P-val	OR	95% C.I.	P-val	OR	95% C.I.	P-val	LATE vs ALS/FTLD	LATE vs Other	ALS/FTLD vs Other
**Clinical Diagnosis**												
Dementia	2.6	[1.1,6.2]	0.034	4.1	[3.1,5.5]	<0.001	3.1	[1.7,5.4]	<0.001	0.795	0.736	0.987
Clinical AD	1.2	[0.6,2.3]	0.605	3.4	[2.6,4.4]	<0.001	0.4	[0.3,0.5]	<0.001	<0.001	0.013	0.012
PPA Diagnosis	1.9	[0.6,5.8]	0.285	0.7	[0.4,1.2]	0.175	4.5	[2.9,7]	<0.001	<0.001	0.391	0.443
bvFTD Diagnosis	2.5	[1,6.8]	0.062	0.6	[0.3,1]	0.066	4.8	[3.2,7.2]	<0.001	<0.001	0.041	0.610
**Degenerative NC**												
HS-A	6.6	[3,14.2]	<0.001	6.4	[4.7,8.9]	<0.001	10.9	[7.1,16.8]	<0.001	0.059	>0.999	0.598
FTLD-Tau	3.3	[1.6,6.5]	<0.001	0.5	[0.4,0.7]	<0.001	0.6	[0.4,0.9]	0.018	0.983	<0.001	<0.001
ADNC	1.5	[0.7,3]	0.295	3.2	[2.4,4.1]	<0.001	0.2	[0.1,0.2]	<0.001	<0.001	0.179	<0.001
Lewy Bodies	1.0	[0.5,1.9]	0.995	2.0	[1.6,2.4]	<0.001	0.3	[0.2,0.5]	<0.001	<0.001	0.212	0.034
**Global Vascular NC**												
CAA	1.0	[0.5,2]	0.947	1.5	[1.2,1.8]	<0.001	0.4	[0.2,0.6]	<0.001	<0.001	0.726	0.058
Atherosclerosis	0.7	[0.3,1.5]	0.388	1.2	[0.9,1.5]	0.186	1.2	[0.8,1.7]	0.398	>0.999	0.592	0.639
Arteriolosclerosis	1.5	[0.7,3.3]	0.259	1.3	[1,1.7]	0.020	1.3	[0.9,1.8]	0.244	0.995	0.978	0.960
**Lesion Vascular NC**												
Infarcts	0.6	[0.2,1.6]	0.323	0.8	[0.6,1.1]	0.160	0.5	[0.2,1]	0.037	0.477	0.943	0.977
Microinfarcts	0.6	[0.3,1.4]	0.219	1.0	[0.8,1.2]	0.809	0.6	[0.4,1]	0.042	0.267	0.660	>0.999
Hemorrhages	1.4	[0.5,4]	0.577	0.7	[0.5,1.1]	0.095	0.6	[0.3,1.4]	0.257	0.997	0.636	0.661

	Other TDP-43			LATE-NC			ALS/FTLD-TDP			Posthoc Tests		
Variable	OR	95% C.I.	P-val	OR	95% C.I.	P-val	OR	95% C.I.	P-val	LATE vs ALS/FTLD	LATE vs Other	ALS/FTLD vs Other
**Gross Atrophy**												
Hippocampal	2.0	[1,4.1]	0.051	2.5	[2,3.2]	<0.001	3.2	[2.2,4.7]	<0.001	0.659	0.919	0.634
Cortical	1.6	[0.8,3.4]	0.184	1.6	[1.2,2]	<0.001	4.0	[2.6,6.2]	<0.001	<0.001	>0.999	0.152
Lobar	1.1	[0.4,3.1]	0.817	1.0	[0.8,1.4]	0.836	9.2	[5.8,14.7]	<0.001	<0.001	0.998	<0.001

Supplementary Table 2. Participant characteristics for ALS/FTLD-TDP group split by those with ALS-TDP only, FTLD-TDP only, or both. Abbreviations: ALS-TDP: amyotrophic lateral sclerosis with TDP-43. FTLD-TDP: frontotemporal lobar degeneration with TDP-43. MCI: mild cognitive impairment. CDR-SB: Clinical Dementia Rating sum-of-the-boxes. AD: Alzheimer's disease. PPA: primary progressive aphasia. bvFTD: behavioral variant of frontotemporal dementia. HS-A: hippocampal sclerosis of aging. ADNC: Alzheimer's disease neuropathologic change. CAA: cerebral amyloid angiopathy.

	**ALS/FTLD-TDP Groups**			**p-values**		
**Characteristic**	**FTLD-TDP Only**, N=146[1]	**ALS-TDP Only**, N=10[1]	**Both ALS-TDP & FTLD-TDP**, N=29[1]	**ALS-TDP Only vs FTLD-TDP Only**[2]	**Both vs FTLD-TDP Only**[2]	**Both vs ALS-TDP Only**[2]
Age at Death (Years)	72.8 (10.5)	72.0 (16.2)	64.7 (9.3)	0.8	<0.001	0.3
Sex				>0.9	0.2	0.6
Female	72 (49%)	5 (50%)	10 (34%)			
Male	74 (51%)	5 (50%)	19 (66%)			
Education (Years)	15.8 (2.8)	16.0 (2.6)	15.9 (3.8)	0.9	0.6	0.9
Missing	3	1	2			
Interval Last Visit to Death (Years)	2.0 (2.2)	1.9 (1.3)	1.0 (0.8)	0.4	0.050	0.026
APOE e4				0.4	0.5	0.8
Absent	83 (65%)	7 (88%)	18 (75%)			
Present	44 (35%)	1 (12%)	6 (25%)			
Missing	19	2	5			
Hereditary FTD Mutation				>0.9	0.061	0.5
Yes	29 (20%)	2 (20%)	11 (38%)			
No or unknown	117 (80%)	8 (80%)	18 (62%)			
TDP-43 Antibody				>0.9	0.6	0.7
Phospho-specific	92 (63%)	7 (70%)	16 (55%)			
Non-phospho-specific	54 (37%)	3 (30%)	13 (45%)			
Other	0 (0%)	0 (0%)	0 (0%)			
Cognitive Status				<0.001	0.079	<0.001
Normal	0 (0%)	7 (70%)	1 (3.4%)			
MCI/Impaired	5 (3.4%)	1 (10%)	1 (3.4%)			
Dementia	141 (97%)	2 (20%)	27 (93%)			
CDR-SB	13.2 (5.3)	2.8 (5.4)	10.8 (4.9)	<0.001	0.009	<0.001
Clinical AD				<0.001	0.013	<0.001
No Impairment	0 (0%)	7 (70%)	1 (3.4%)			
Not AD	99 (68%)	3 (30%)	24 (83%)			

Characteristic	ALS/FTLD-TDP Groups: FTLD-TDP Only, N=146[1]	ALS-TDP Only, N=10[1]	Both ALS-TDP & FTLD-TDP, N=29[1]	p-values: ALS-TDP Only vs FTLD-TDP Only[2]	Both vs FTLD-TDP Only[2]	Both vs ALS-TDP Only[2]
AD	47 (32%)	0 (0%)	4 (14%)			
PPA Diagnosis				0.6	0.093	>0.9
Absent	91 (64%)	7 (78%)	23 (82%)			
Present	52 (36%)	2 (22%)	5 (18%)			
Missing	3	1	1			
bvFTD Diagnosis				0.046	<0.001	<0.001
Absent	93 (64%)	10 (100%)	7 (24%)			
Present	53 (36%)	0 (0%)	22 (76%)			
HS-A				0.042	0.010	0.7
Absent	92 (63%)	10 (100%)	26 (90%)			
Present	54 (37%)	0 (0%)	3 (10%)			
FTLD-Tau				>0.9	0.8	>0.9
Absent	122 (84%)	8 (80%)	23 (79%)			
Present	24 (16%)	2 (20%)	6 (21%)			
ADNC				0.6	0.037	0.035
None/Low	112 (78%)	5 (62%)	28 (97%)			
Intermediate/High	32 (22%)	3 (38%)	1 (3.4%)			
Missing	2	2	0			
CAA				0.15	0.7	0.14
None/Mild	128 (89%)	6 (67%)	27 (93%)			
Moderate/Severe	16 (11%)	3 (33%)	2 (6.9%)			
Missing	2	1	0			
Lewy Bodies				>0.9	0.6	>0.9
Absent	122 (84%)	8 (89%)	26 (90%)			
Present	24 (16%)	1 (11%)	3 (10%)			
Missing	0	1	0			
Atherosclerosis				0.8	0.047	0.10
None/Mild	101 (70%)	6 (60%)	26 (90%)			
Moderate/Severe	44 (30%)	4 (40%)	3 (10%)			
Missing	1	0	0			
Arteriolosclerosis				0.5	0.14	>0.9
None/Mild	70 (50%)	6 (67%)	19 (68%)			
Moderate/Severe	69 (50%)	3 (33%)	9 (32%)			

Characteristic	ALS/FTLD-TDP Groups<br>FTLD-TDP Only, N=146[1]	ALS-TDP Only, N=10[1]	Both ALS-TDP & FTLD-TDP, N=29[1]	p-values<br>ALS-TDP Only vs FTLD-TDP Only[2]	Both vs FTLD-TDP Only[2]	Both vs ALS-TDP Only[2]
Missing	7	1	1			
Infarcts				>0.9	0.9	>0.9
Absent	137 (94%)	10 (100%)	28 (97%)			
Present	9 (6.2%)	0 (0%)	1 (3.4%)			
Microinfarcts				0.5	0.8	0.4
Absent	127 (87%)	10 (100%)	24 (83%)			
Present	19 (13%)	0 (0%)	5 (17%)			
Hemorrhages				0.8	0.6	0.5
Absent	136 (96%)	7 (88%)	28 (100%)			
Present	6 (4.2%)	1 (12%)	0 (0%)			
Missing	4	2	1			
Gross Hippocampal Atrophy				0.10	0.009	>0.9
None/Mild	37 (28%)	5 (62%)	13 (59%)			
Moderate/Severe	94 (72%)	3 (38%)	9 (41%)			
Missing	15	2	7			
Gross Cortical Atrophy				<0.001	<0.001	0.3
None/Mild	19 (17%)	6 (86%)	10 (56%)			
Moderate/Severe	96 (83%)	1 (14%)	8 (44%)			
Missing	31	3	11			
Gross Lobar Atrophy				0.055	0.057	0.8
Absent	40 (34%)	6 (75%)	11 (61%)			
Present	76 (66%)	2 (25%)	7 (39%)			
Missing	30	2	11			

[1] Mean (SD); n (%)

[2] Wilcoxon rank sum test; Pearson's Chi-squared test

SUPPLEMENTARY FIGURES

Supplementary Figure 1. Sankey diagram showing NACC participants who were either lost to follow-up or died, split by etiologic diagnosis categories at last available visit. Depicts participants who were noted as deceased ("death reported") and those who eventually went on to neuropathological assessment ("autopsy"). Numbers below represent the number of participants who made it at least to that stage, with percentage of previous. Total numbers are shown at the top for death reported and autopsy. Abbreviations: NACC: National Alzheimer's Coordinating Center. AD: Alzheimer's disease. FTLD: frontotemporal lobar degeneration. CBD: corticobasal degeneration. PSP: progressive supranuclear palsy. LB: Lewy body dementia. No Imp.: No impairment. Diag. N/A: etiologic diagnosis not available at last visit.

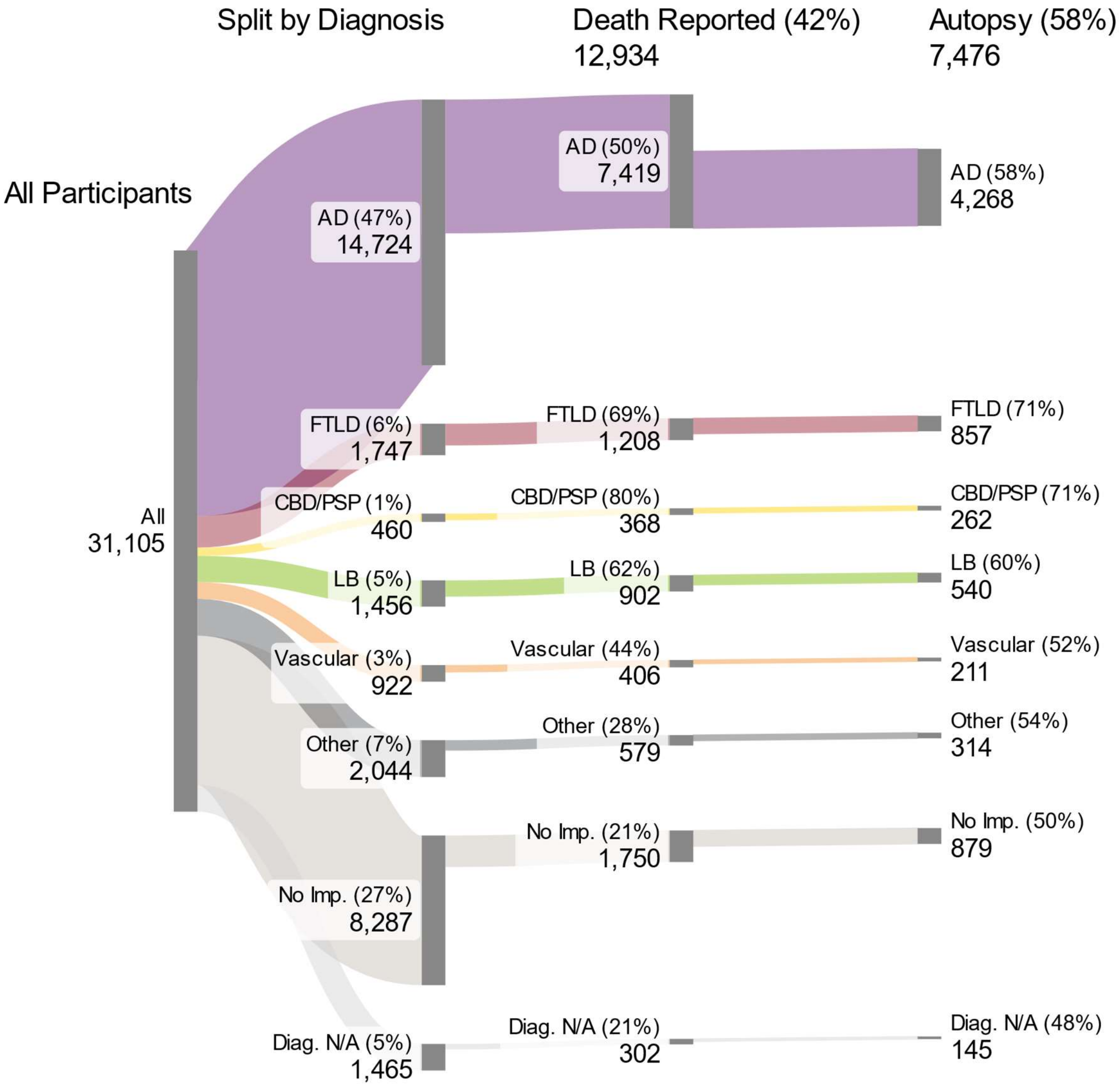

Supplementary Figure 2. Sankey diagram showing NACC participants with version 10 or higher neuropathology forms, by whether or not they were assessed for TDP-43, and whether they had all TDP-43 measures available, split by etiologic diagnosis categories at last available visit. Numbers below represent the number of participants who made it at least to that stage. The "split diagnosis" percentage is a percentage of the total, while the "Staining for TDP-43" and "All TDP-43 Meas." are percentages with respect to all participants with that particular etiologic diagnosis. Total numbers are shown at the top, showing that 84% of v10+ participants were stained for TDP-43, and 50% of participants had all TDP-43 related measures (and are the subset used in **Part II**). Abbreviations: NACC: National Alzheimer's Coordinating Center. AD: Alzheimer's disease. FTLD: frontotemporal lobar degeneration. CBD: corticobasal degeneration. PSP: progressive supranuclear palsy. LB: Lewy body dementia. No Imp.: No impairment. Diag. N/A: etiologic diagnosis not available at last visit.

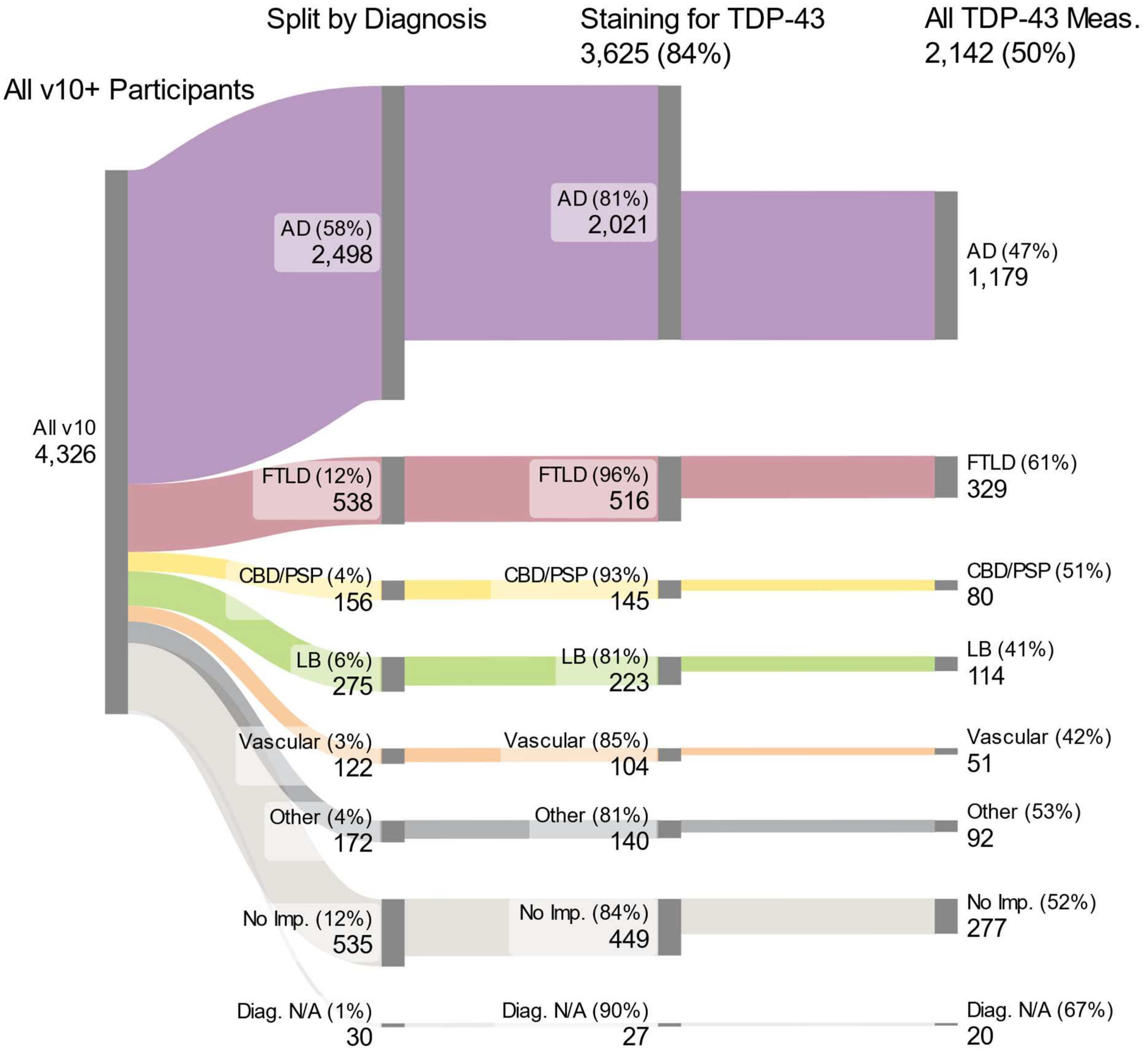

Supplementary Figure 3. Availability of TDP-43 related pathology measures and presence by Alzheimer's Disease Center (ADC). **a.** Availability of TDP-43 related pathology information by ADC. **b.** Presence of TDP-43 related pathologies by ADC. ADC variable is randomly generated for anonymity. ADCs ordered by number of total participants with v10+ pathology forms. Abbreviations: FTLD-TDP: frontotemporal lobar degeneration with TDP-43 pathology. ALS: amyotrophic lateral sclerosis. Amyg.: amygdala. Hipp.: hippocampus. EC/ITC: Entorhinal cortex/inferior temporal cortex. NeoC: neocortex. SC: spinal cord. HS-A: hippocampal sclerosis of aging.

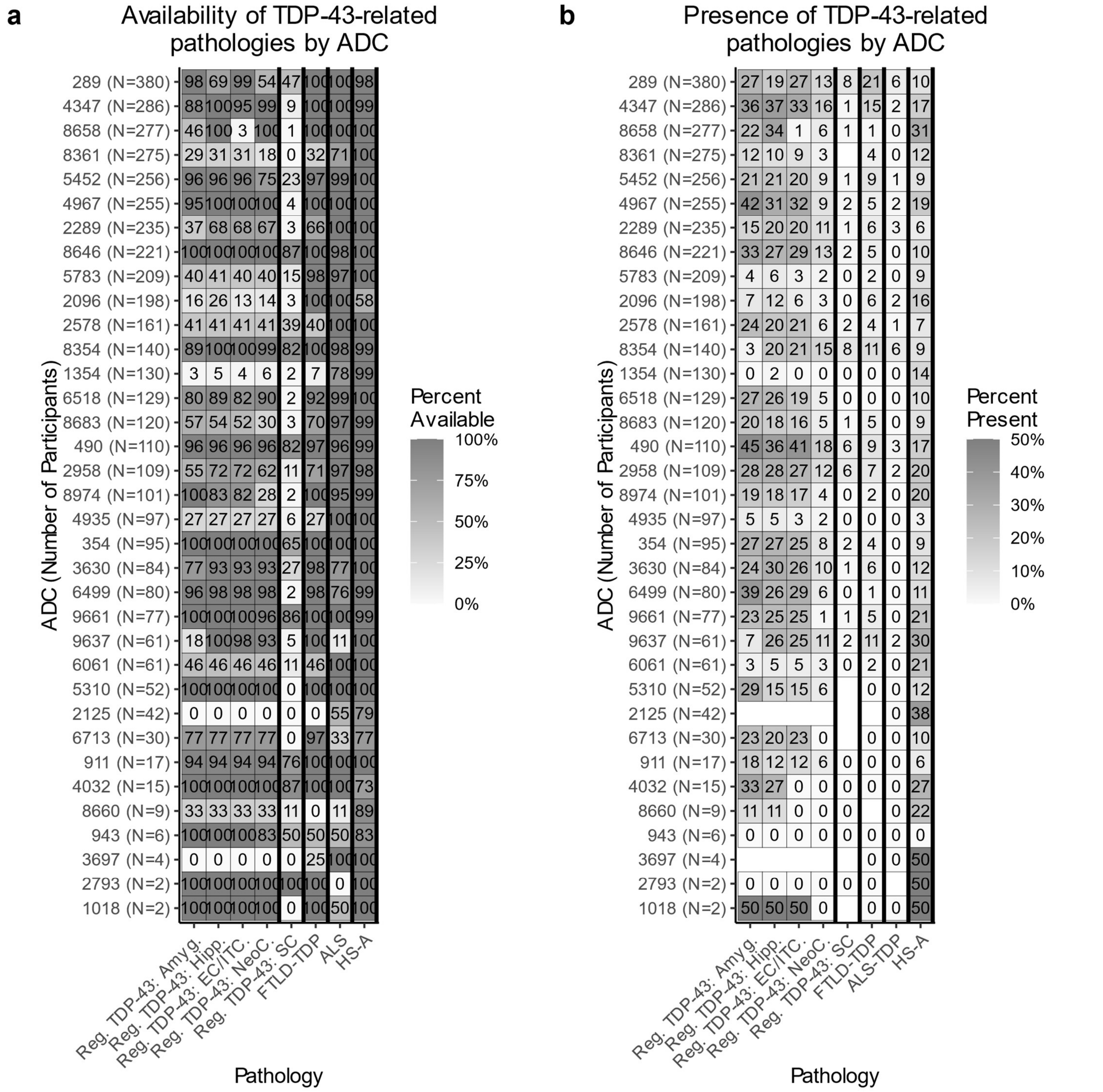

Supplementary Figure 4. TDP-43 group classification by Alzheimer's Disease Center (ADC). **a.** Percentage of participants by ALS/FTLD-TDP, LATE-NC, Other TDP-43, or no brain TDP-43, by ADC. **b**. Percentage of participants with LATE-NC by stage. ADC variable is randomly generated for anonymity. ADCs ordered by number of total participants contributed. Abbreviations: FTLD-TDP: Frontotemporal lobar degeneration with TDP-43 pathology. ALS: Amyotrophic lateral sclerosis. LATE-NC: Limbic-predominant age-related TDP-43 encephalopathy.

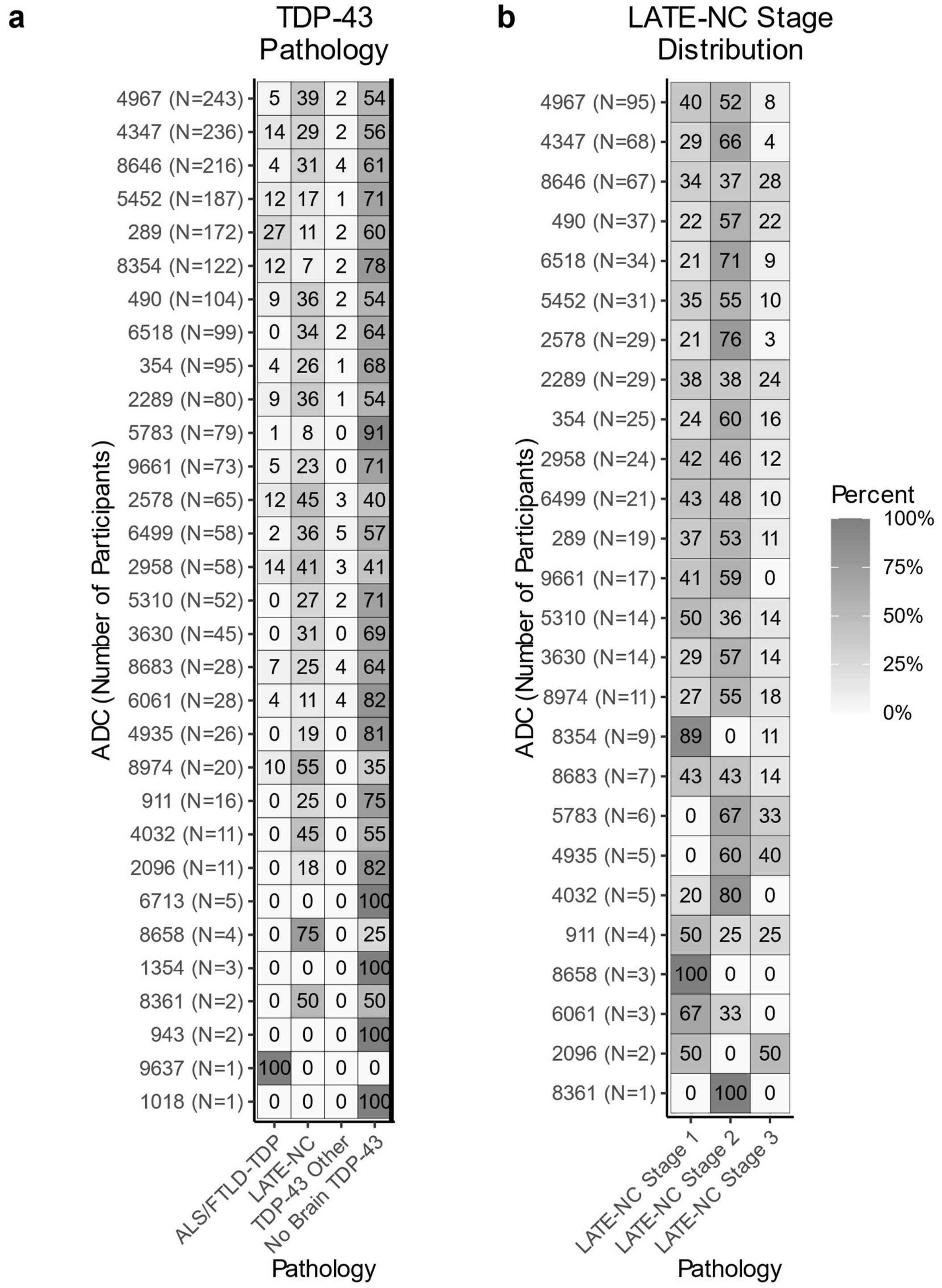

Supplementary Figure 5. Histograms for distributions of ages at death by TDP-43 neuropathology categories. For y-axes, note each graph is in a different scale. For x-axes, line at 85 years old to help demarcate younger-old from oldest-old (i.e. 85 or older) participants. Abbreviations: LATE-NC: limbic-predominant age-related TDP-43 encephalopathy neuropathologic change. ALS/FTLD-TDP: amyotrophic lateral sclerosis or frontotemporal lobar degeneration with TDP-43 pathology.

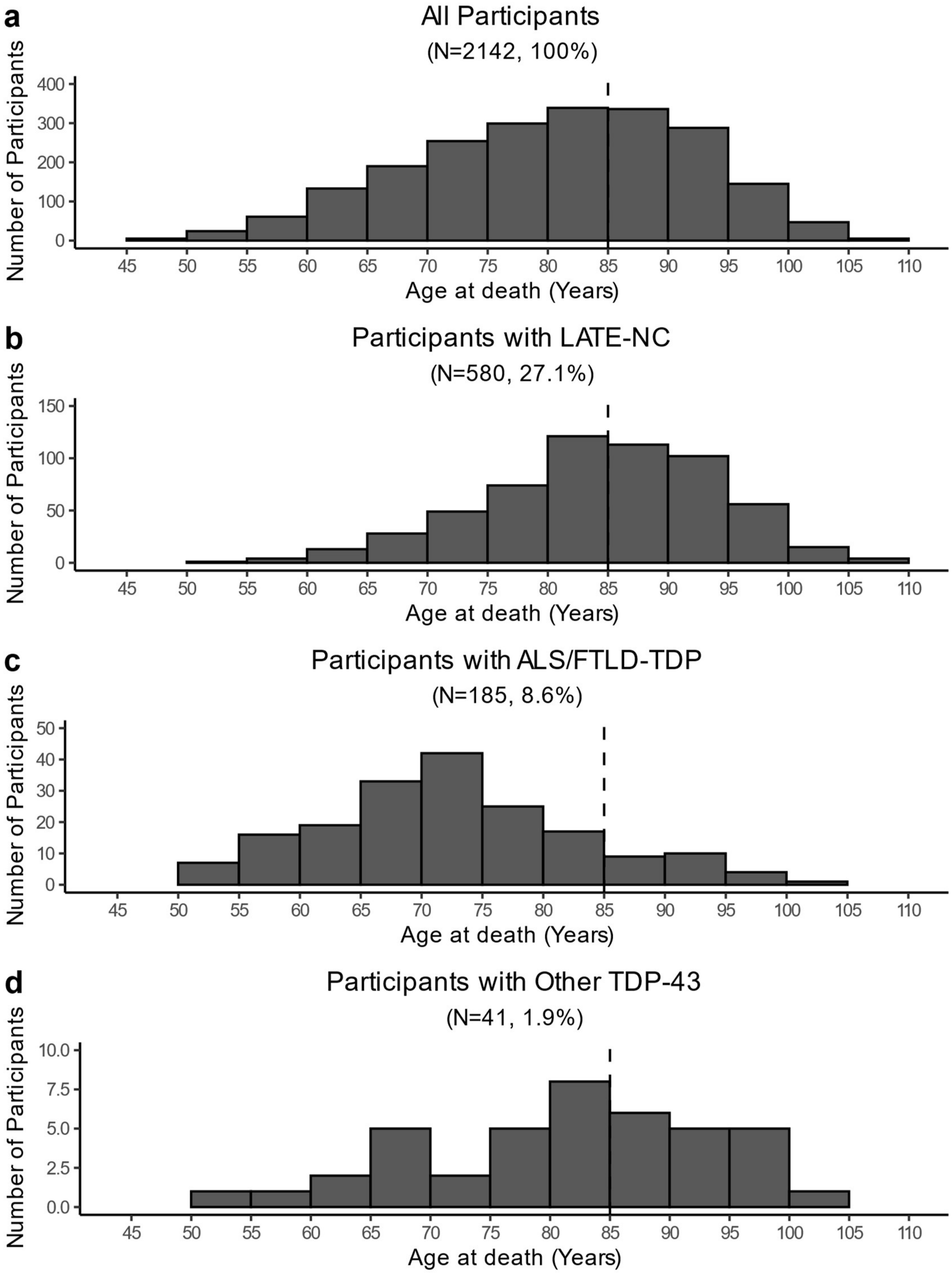

Supplementary Figure 6. Odds ratios for various clinical diagnoses and other pathologies with regards to TDP-43 categories, ALS/FTLD-TDP, LATE-NC, and Other TDP-43, compared to those without TDP-43. Multilevel logistic regressions with varying intercepts by center, adjusted for age at death, sex, education, and interval between last visit and death. Dots represent odds ratios and error bars represent 95% confidence intervals. Abbreviations: LATE-NC: limbic-predominant age-related TDP-43 encephalopathy neuropathologic change. ALS/FTLD-TDP: amyotrophic lateral sclerosis or frontotemporal lobar degeneration (FTLD) with TDP-43 pathology. AD: Alzheimer's disease. PPA: primary progressive aphasia. bvFTD: behavioral variant of frontotemporal dementia. HS-A: hippocampal sclerosis of aging. ADNC: Alzheimer's disease neuropathologic change. CAA: cerebral amyloid angiopathy.

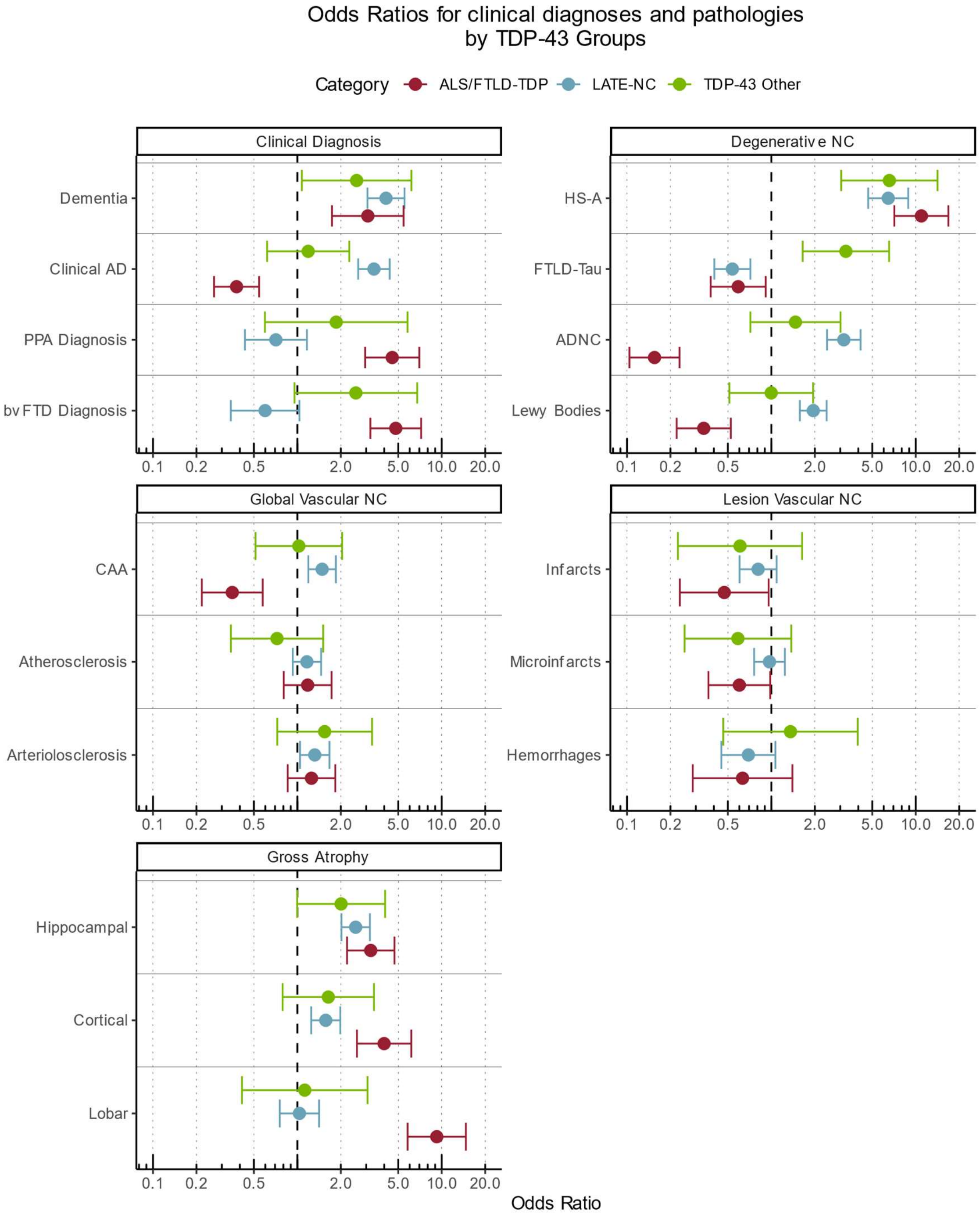

Supplementary Figure 7. Venn diagrams for presence of regional TDP-43 inclusions by pathological category. Presence of TDP-43 inclusions by region for participants with **a.** ALS-TD only, **b.** FTLD-TDP only, and **c.** both ALS-TDP and FTLD-TDP. Abbreviations: ALS-TDP: amyotrophic lateral sclerosis with TDP-43. FTLD-TDP frontotemporal lobar degeneration with TDP-43 pathology. Amyg.: amygdala. Hipp.: hippocampus. EC/ITC: entorhinal cortex/inferior temporal cortex. NeoC: neocortex.

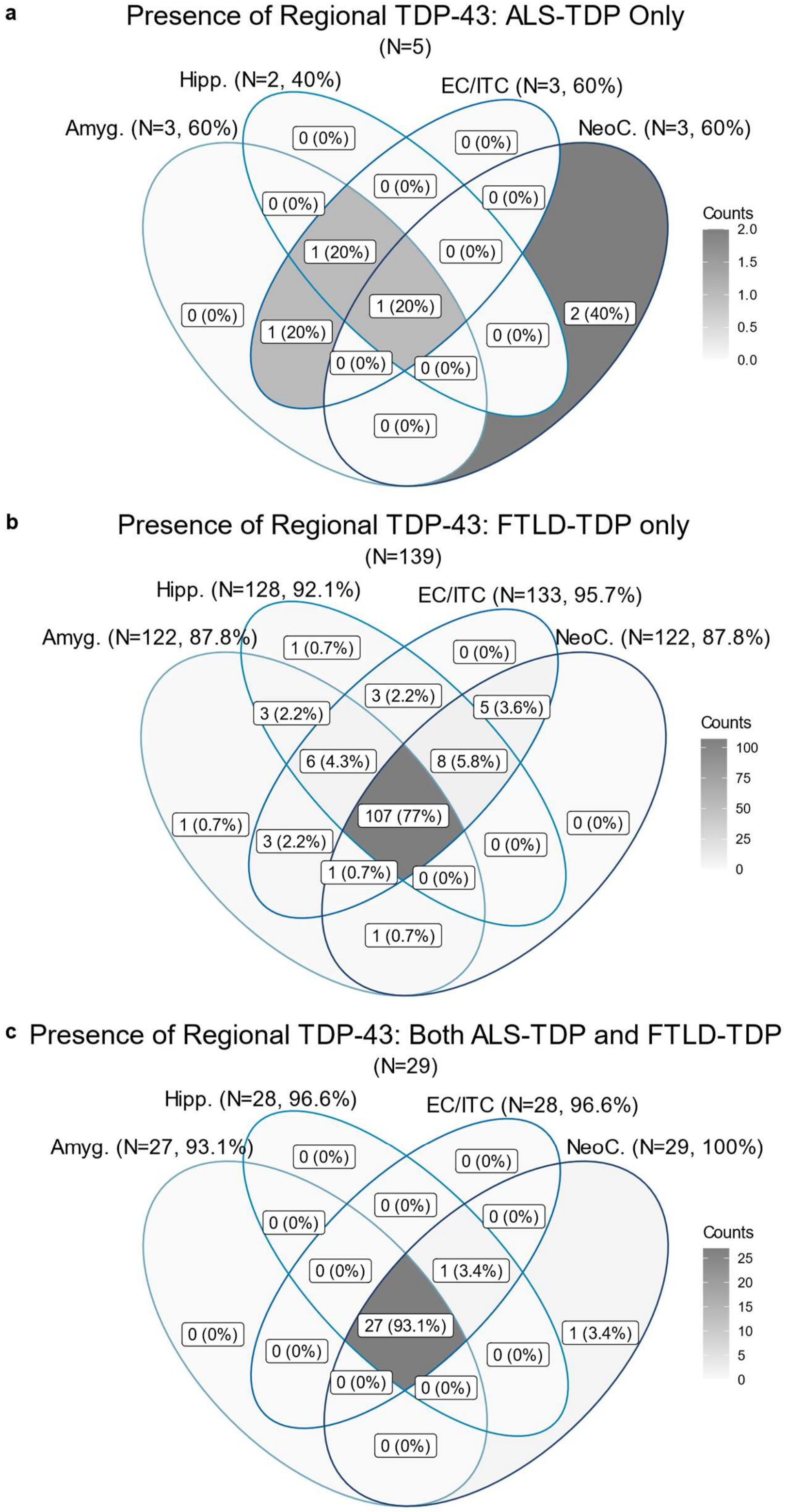

Supplement: Supplementary file 1 — Supplementary file1 (PDF 4536 KB) [file 401_2024_2728_MOESM1_ESM.pdf]
